# Genome-wide identification of bacterial plant colonization genes

**DOI:** 10.1371/journal.pbio.2002860

**Published:** 2017-09-22

**Authors:** Benjamin J. Cole, Meghan E. Feltcher, Robert J. Waters, Kelly M. Wetmore, Tatiana S. Mucyn, Elizabeth M. Ryan, Gaoyan Wang, Sabah Ul-Hasan, Meredith McDonald, Yasuo Yoshikuni, Rex R. Malmstrom, Adam M. Deutschbauer, Jeffery L. Dangl, Axel Visel

**Affiliations:** 1 US Department of Energy Joint Genome Institute, Walnut Creek, California, United States of America; 2 Department of Biology, Department of Microbiology and Immunology, Curriculum in Genetics and Molecular Biology, Howard Hughes Medical Institute, Carolina Center for Genome Sciences, University of North Carolina at Chapel Hill, Chapel Hill, North Carolina, United States of America; 3 Environmental Genomics and Systems Biology Division, Lawrence Berkeley National Laboratory, Berkeley, California, United States of America; 4 School of Natural Sciences, University of California Merced, Merced, California, United States of America; Duke University, United States of America

## Abstract

Diverse soil-resident bacteria can contribute to plant growth and health, but the molecular mechanisms enabling them to effectively colonize their plant hosts remain poorly understood. We used randomly barcoded transposon mutagenesis sequencing (RB-TnSeq) in *Pseudomonas simiae*, a model root-colonizing bacterium, to establish a genome-wide map of bacterial genes required for colonization of the *Arabidopsis thaliana* root system. We identified 115 genes (2% of all *P*. *simiae* genes) with functions that are required for maximal competitive colonization of the root system. Among the genes we identified were some with obvious colonization-related roles in motility and carbon metabolism, as well as 44 other genes that had no or vague functional predictions. Independent validation assays of individual genes confirmed colonization functions for 20 of 22 (91%) cases tested. To further characterize genes identified by our screen, we compared the functional contributions of *P*. *simiae* genes to growth in 90 distinct in vitro conditions by RB-TnSeq, highlighting specific metabolic functions associated with root colonization genes. Our analysis of bacterial genes by sequence-driven saturation mutagenesis revealed a genome-wide map of the genetic determinants of plant root colonization and offers a starting point for targeted improvement of the colonization capabilities of plant-beneficial microbes.

## Author summary

Plants fix carbon to create an abundance of sugars and amino acids, thus providing an enticing environment for microorganisms that reside in soil. Once these microorganisms have colonized the root environment, they can dramatically influence plant growth and development. We set out to identify a comprehensive set of microbial genes that control or influence root colonization, using a genome-wide transposon mutagenesis approach (randomly barcoded transposon sequencing [RB-TnSeq]). By using this method, we identified several hundred genes that, when mutated, affect the ability of the bacterium *P*. *simiae* to competitively colonize the root system of the model plant *A*. *thaliana*. These included many genes purported to be involved in carbohydrate metabolism, cell wall biosynthesis, and motility, underscoring the notion that sugar metabolism, defense, and motility are all key features of a root-colonizing microbe. We also identified several amino acid transport and metabolism genes with mutations that confer a fitness advantage in root colonization. Lastly, we identified several genes with no known function that significantly alter root colonization ability when mutated. These findings suggest novel engineering strategies to improve biological product development, and will facilitate the mechanistic exploration of the root colonization process.

## Introduction

Plant health is intimately influenced by a diverse community of microorganisms inhabiting the root surface (rhizoplane) and endophytic compartment [1]. This root microbiome is recruited from surrounding soil communities [2–4] and is thought to be modulated by host plant immune function, root exudate-mediated signaling and metabolic compatibility, as well as intermicrobial interactions within the rhizosphere [5–7]. These interactions, especially during the initial colonization period, are critical for establishment of a root-associated bacterial community that is distinct from that of the surrounding soil. Extensive studies of plant pathogens have established the role of plant genetic factors, including immune phytohormone pathways, in controlling the ability of bacteria to colonize plants [5,8–10]. Although there is increasing recognition that root microbiomes, in particular plant growth-promoting rhizobacteria (PGPR), may be harnessed to improve plant fitness in agricultural applications, progress toward this goal requires a more thorough understanding of the bacterial genetic factors contributing to root colonization and fitness in the root microbiome [11].

Root-associated bacterial communities have been defined for several plants, including *A*. *thaliana*, using culture-independent 16S rRNA sequencing strategies [3,4]. Bacterial communities across diverse plant species show similar dominant representation of Proteobacteria, Actinobacteria, and Bacteroidetes phyla [1]. The Pseudomonadaceae (within the Proteobacteria phylum), in particular, comprise many genera capable of plant association, with the best studied examples (e.g., *Pseudomonas fluorescens* and *P*. *syringae*) being commensals or pathogens, respectively [12,13]. Many other isolates within the Pseudomonadaceae family are characterized as PGPR, which can enhance plant growth and viability through beneficial immune stimulation (induced systemic resistance [ISR]) [14], by improvement of soil nutrient acquisition, or by directly triggering plant growth pathways through phytohormone production [1,15]. Additionally, *P*. *fluorescens* spp. have been shown to actively protect crops from a variety of fungal pathogens [16]. *P*. *simiae* WCS417r was originally characterized as a biocontrol isolate on wheat [17]. This strain was originally characterized as a member of the *P*. *fluorescens* group but was reclassified based on its genome sequence homology to the *P*. *simiae*-type strain [18] and is a well-studied example of a PGPR [18]. WCS417r displays other PGPR activities, including ISR induction, siderophore production, lateral root growth stimulation, and activation of auxin signaling pathways [19]. Importantly, WCS417r can colonize the roots of many plant species including *Arabidopsis* [20]. These features make colonization of *Arabidopsis* roots by WCS417r an ideal system for identifying generalized bacterial colonization traits.

Conventional, nonsaturation screens of transposon mutagenesis libraries of *P*. *fluorescens* and *P*. *putida* strains led to the identification of genes required for root and rhizosphere colonization [12,21]. To enable the generation of a comprehensive genome-wide map of root colonization genes, we used randomly barcoded transposon mutagenesis sequencing (RB-TnSeq), a barcode-enabled extension of transposon mutagenesis coupled to high-throughput sequencing (transposon mutagenesis sequencing [TnSeq]) [22] that allows for the generation of reusable libraries of unique, mapped, and barcoded insertion mutant strains [23,24]. We adopted RB-TnSeq to construct a genome-wide map of *P*. *simiae* WCS417r plant-association factors in an in vivo screen using *Arabidopsis* as the host plant. This screen revealed mutations in 115 genes that have a negative impact on the ability of *P*. *simiae* WCS417r to colonize roots. In addition to genes linked to well-known colonization traits such as motility and carbon metabolism, our mutant screen revealed additional, previously uncharacterized genes. Our screen also identified 243 genes, the loss of function of which enhances colonization fitness. Many of the genes identified in each class are clustered into predicted operons. Integration of the genome-wide colonization data with RB-TnSeq phenotypes from more than 90 different in vitro growth conditions [23] highlighted motility, stress response, amino acid metabolism, as well as potentially unknown pathways as being functionally important for root/bacterial interactions.

## Results

### A genome-wide map of plant colonization genes

To enable the generation of a genome-wide map of genes required for plant root colonization in *P*. *simiae*, we used RB-TnSeq with a mariner transposon to create a saturation mutagenesis library of *P*. *simiae* WCS417r [22–25]. We selected WCS417r based on its plant growth promoting potential, its ease of transformation at high efficiency, and its tractability for lab manipulation. By high-throughput sequence analysis of barcoded insertion mutants, we identified and mapped 110,142 unique transposon insertion sites, distributed throughout the genome [18] at an average of approximately 18 insertions per 1,000 bp (S1 Fig, S1 Data). Most insertions (59.5%) mapped to a gene body, with 84% of genes harboring at least 1 insertion event (median insertions per gene: 9; S1 Fig). Of the remaining 827 genes with no insertion mutant detected, nearly half shared significant homology (Materials and methods) to genes known to be essential in other species (385; 55.6% of such genes in the WCS417r genome; S1 Fig), suggesting that insertions in these genes are lethal in *P*. *simiae*. Furthermore, 146 of the untargeted genes contained fewer than 3 potential thymine-adenine dinucleotide mariner transposon insertion sites, representing 59% of such genes in the WCS417r genome. Thus, our library includes null mutations in the vast majority of nonessential genes in the *P*. *simiae* WCS417r genome, supporting its utility for large-scale genetic screening for various phenotypes.

**Fig 1:**
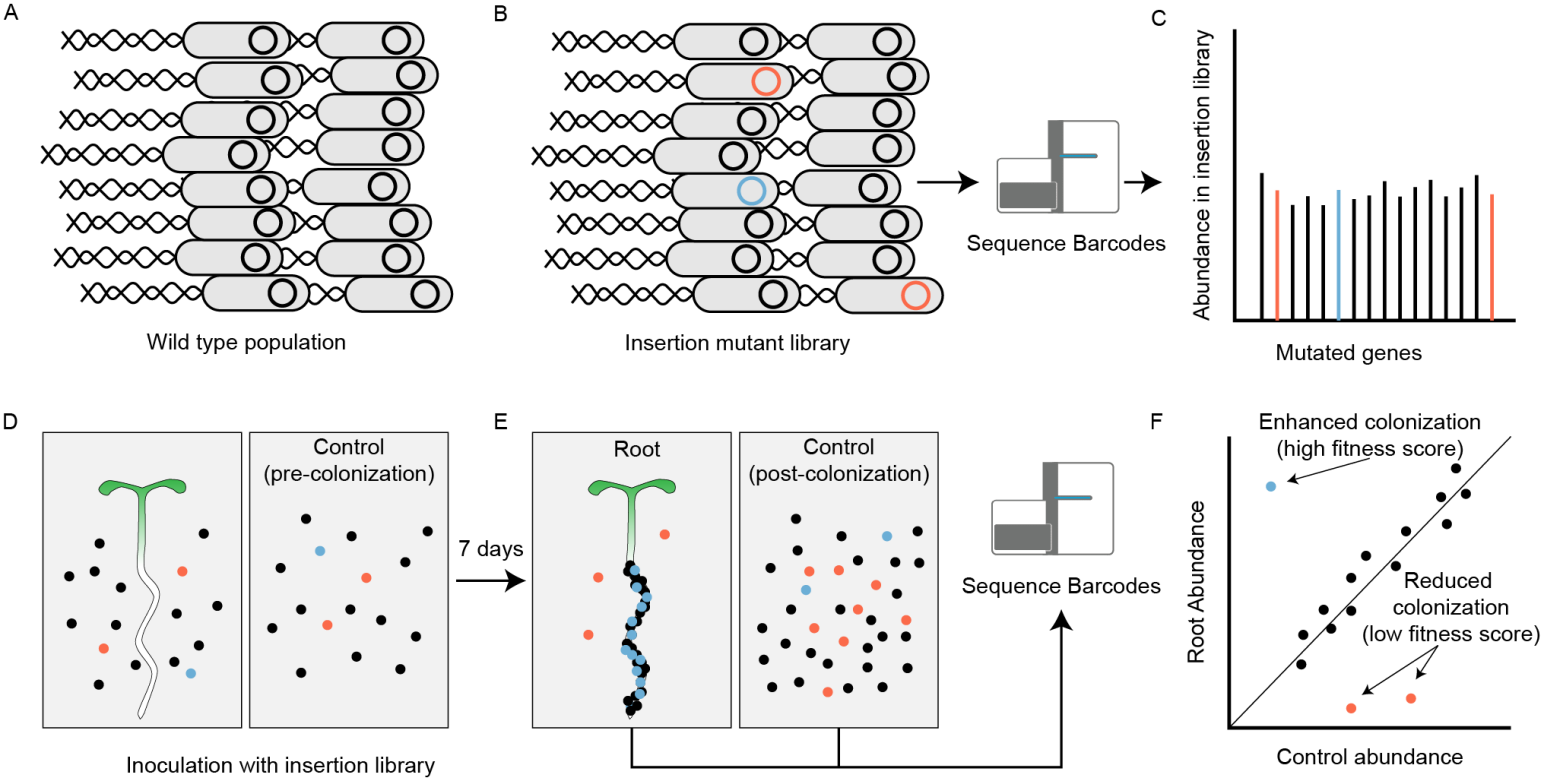
Overview of root colonization randomly barcoded transposon mutagenesis sequencing (RB-TnSeq) screen. Wild-type *Pseudomonas simiae* WCS417r (A) was mutagenized with a mariner transposon system, generating 110,142 insertion mutants (B). Most insertion mutations do not significantly alter the growth phenotype on the root (black), while some insertion mutations make these mutant strains more (blue) or less (red) likely to colonize plant roots, while not significantly affecting their ability to grow in liquid culture or on the nylon substrate in the absence of plants (C). This mutant strain library was exposed to vertically-oriented phytagel plates with (D, left) or without (D, right) *Arabidopsis thaliana* seedlings. After colonization, surviving mutant strains from the root and from the plant-free mesh were collected and the abundance of insertion mutant strains within each population was quantified by RB-TnSeq. (F) Genes with under-represented insertion counts in the root population compared with the control population (shown here in red) were given low fitness scores (efficient colonization), while genes with over-represented counts were given high fitness scores.

To determine which genes are necessary for root association, we designed a competitive colonization screen in which *P*. *simiae* mutant strains migrate from support medium through a porous nylon filter toward the root system of *Arabidopsis* seedlings, where they can attach and propagate (Fig 1 and S2 Fig). After a colonization period and removal of loosely adhering bacteria, root-associated bacteria were isolated as a combined rhizoplane and endophytic sample. Controls required for data analysis included a “no root initial” (NRI) sample (i.e., an empty nylon filter incubated on a plate containing the mutant library, harvested the same day as the library was inoculated) and a “no root final” (NRF) sample (i.e., a filter incubated on the plate in the absence of plants for a full week). In total, we analyzed samples and controls harvested from approximately 15,000 seedlings. We sequenced approximately 181,300 unique barcodes from each pooled root sample (see Materials and methods), corresponding to approximately 240 colonization events per individual root. Of all barcode sequence reads, we mapped 70% to known barcoded insertion sites.

We used sequenced barcode read counts to quantify the representation of mutants in each sample and compared barcode frequencies across samples [24] (Fig 1; Materials and methods). After normalization of total counts across samples, we determined 3 separate derived fitness scores for each gene. Each of these scores measures a different potential effect influencing microbial growth in these experimental conditions: a “mesh fitness score” comparing the NRF and NRI samples and thus measuring changes in the ability to growth on the nylon mesh alone; a “root + mesh fitness score” comparing the “root” and the NRI samples, which measures the overall ability to grow on the root and the nylon mesh; and a “root fitness score,” comparing the root and NRF samples directly, which represents the “root + mesh fitness score” corrected for the “mesh fitness score” to quantify the ability to grow on the root after correction for mesh-related effects.

We used this root fitness score (Materials and methods) to identify mutant strains corresponding to 358 genes as significantly depleted or enriched in the root-associated sample, which included 115 colonization-depleted genes (that, when mutated, results in reduced colonization ability) and 243 colonization-enriched genes (that, when mutated, increased colonization ability, S3 Fig).

**Fig 2:**
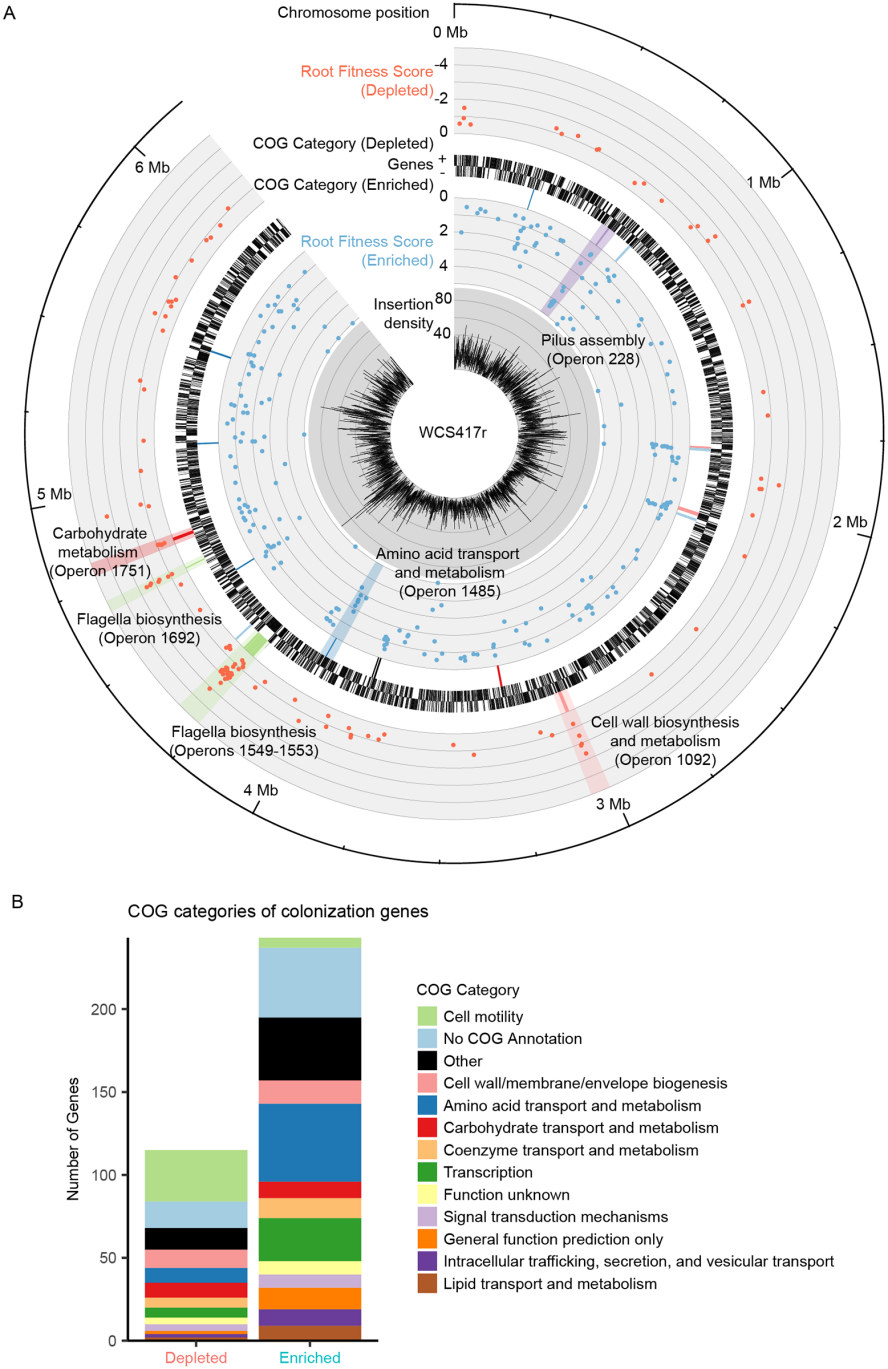
Genome-wide map of root colonization. (A) Inner to outer tracks: transposon insertion density (per 1 kb); fitness score for genes with enhanced colonization ability when mutated; dominant cluster of orthologous group (COG) category for operons with 3 or more colonization-enriched genes (Table 1), gene density (for each strand); dominant COG category for operons with 3 or more colonization-reduced genes (Table 1); fitness score for genes with reduced colonization ability when mutated; chromosomal position. (B) Color legend of dominant COG categories and highlights are shown. (B) Distribution of genes significantly depleted or enriched among COG categories (see S1 Data).

We used the colonization fitness scores of individual genes to create a genome-wide map of the root colonization trait (Fig 2). Genes significantly contributing to colonization fitness were distributed throughout the *P*. *simiae* genome, with many clustering together (Fig 2, Table 1). Strikingly, 45 of the 115 genes mutated in colonization-depleted strains are clustered into 8 predicted operons, each containing at least 3 genes that decrease colonization fitness when mutated (Table 1). Similarly, 62 of the 243 genes mutated in colonization-enriched strains were located within 14 predicted operons containing at least 3 genes that significantly increased colonization fitness when mutated (Table 1). Thus, 22 predicted operons contained 3 or more genes with significant fitness scores corresponding to enhanced or reduced colonization ability. In 21 of these operons, all genes with significant fitness scores contributed to colonization in a consistent direction within the operon, with 14 operons exhibiting >50% of the constituent genes as significant.

**Table 1:** Putative operons containing 3 or more colonization genes. All genes selected for validation in these operons were successfully validated.

Operon ID	Number of Genes	Number Depleted	Number Enriched	Putative Function	Genes Selected for Validation
1,551	15	10	-	Flagellar biosynthesis/regulation	
1,550	9	7	-	Flagellar biosynthesis/regulation	*PS417_19755*
1,549	13	6	-	Chemotaxis	
1,751	10	5	1	Carbohydrate transport/metabolism	*PS417_22145*
1,553	6	5	-	Flagellar biosynthesis/regulation	
1,092	10	5	-	arn/pmr/cell-wall modifying	*PS417_13795*
1,692	5	4	-	Flagellar biosynthesis/regulation	
1,569	4	3	-	Carbohydrate transport/metabolism	
228	8	-	8	Flp pilus assembly locus	*PS417_03095*
622	9	-	7	Cell wall/membrane/envelope biogenesis	*PS417_08225*
742	12	-	5	Cell wall/membrane/envelope biogenesis	*PS417_09620*
127	6	-	5	Amino acid transport/metabolism	*PS417_01565*
1,652	5	-	5	Amino acid transport/metabolism	*PS417_21035*
1,465	4	-	4	Organic acid metabolism	
749	11	-	4	Lipid/carbohydrate/inorganic ion metabolism	*PS417_09700*
621	5	-	4	Cell wall/membrane/envelope biogenesis	*PS417_08165*, *PS417_08190*
2,025	7	-	4	Amino acid transport/metabolism	
1,398	6	-	4	ATP-binding cassette transporter/defense	*pedA (PS417_17485)*
265	11	-	3	General function prediction/Function unknown	
1,395	6	-	3	Energy production/conversion	
1,187	8	-	3	Carbohydrate/lipid transport and metabolism	*PS417_14935*
1,879	5	-	3	Amino acid transport/metabolism	

We examined predicted functions of the identified colonization genes and operons based on clusters of orthologous groups (COG) of proteins annotations [26,27]. Among colonization-depleted genes, motility was the most common COG category (*P* < 1.88 e-20; hypergeometric test), followed by cell wall/membrane/envelope biogenesis (*P* < 2 e-3) and carbohydrate transport and metabolism (*P* < 2.43 e-2; Fig 2), consistent with the known roles of motility, lipopolysaccharide production, and sugar metabolism in root colonization and activity[12]. Among colonization-enriched genes, common COG categories included amino acid metabolism and transport (*P* < 1.38 e-2), cell wall/membrane biogenesis (*P* < 5.99 e-3), and transcription (*P* < 4.18 e-3; Fig 2). Taken together, our genome-wide colonization screen allowed for the simultaneous functional assessment of nearly all genes within the WCS417r genome for their contribution to plant colonization, and we identified a substantial number of genes and operons likely to be important for this process.

### Validation of candidate colonization mutants

**Fig 3:**
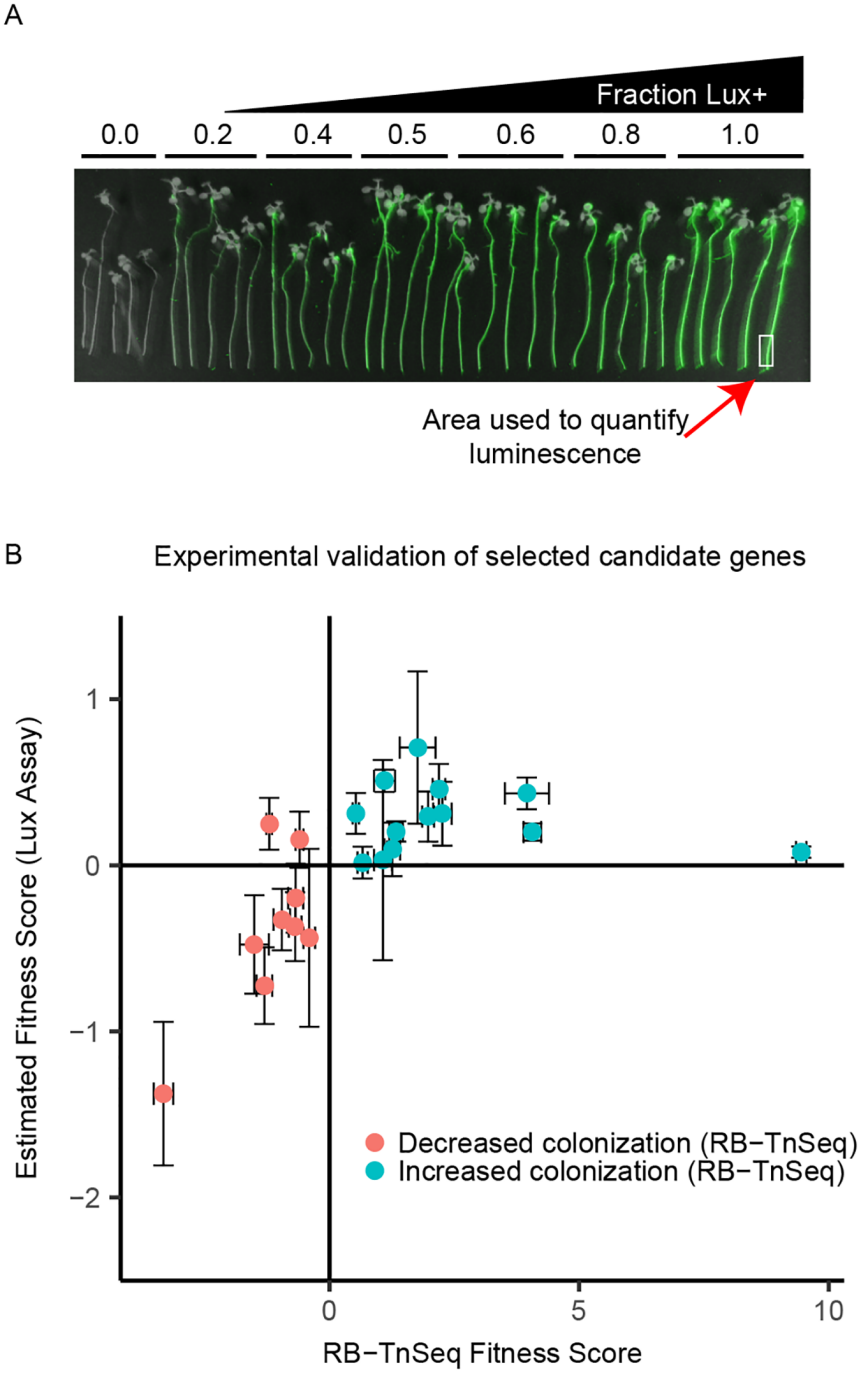
Selected candidates are validated using secondary luciferase-based screen. Twenty-two mutant strains were retrieved from an arrayed clone library for further validation. These strains were competed against an engineered wild-type (WT) *P*. *simiae* strain that produces luciferase. (A) False-color image of competition between the nonengineered WT WCS417r strain versus the luciferase-producing (Lux+) strain. Each group of 5 roots represents a different ratio of Lux+ to WT (0.0–1.0). Luminescence intensity is false colored in green. White rectangle indicates approximate region of the root tip used to measure luciferase activity. (B) All 22 insertion mutant strains (described in S1 Data) were competed with the Lux+ on roots and empty phytagel/mesh plates after inoculation as a 1:1 mixture. A ratio of each mutant strain to the Lux+ strain on the roots was estimated by interpolating the luminescence intensity of the root tip onto a standard curve (S5 Fig, S1 Data). The estimated fitness score (Lux+ colonization index [CI] in S1 Data) was then derived from the log-transformed root ratio minus the log-transformed mesh (no root final [NRF]) ratio (see S1 Data). Error bars are ± standard error (*n* = 3 biological replicates for y-axis, *n* = 15 for x-axis). Abbreviation: RB-TnSeq, randomly barcoded transposon mutagenesis sequencing.

To evaluate the robustness of our screen, we isolated individual insertion mutant strains from sequence-informed WCS417r library arrays (Materials and methods). We selected 22 insertion mutant strains to validate (using a single insertion mutant strain per gene) covering a diversity of potentially interesting putative functions, with some representing operons containing multiple genes with significant fitness scores (Table 1), and others representing individual genes with a broad range of negative or positive fitness score effects (S1 Data). The selected mutants included 9 predicted to have compromised colonization fitness and 13 predicted to have increased colonization ability (S1 Data). We designed a competitive colonization screen in which individual mutants compete against a luminescent, but otherwise wild-type (WT) *P*. *simiae* WCS417r strain, and direct luminescence quantification of roots can be used to measure competitive fitness (Fig 3, Materials and methods).

We observed that 7 out of 9 colonization-depleted insertion mutants were out-competed by the luciferase-producing (Lux+) strain (Fig 3). Similarly, all 13 colonization-enriched insertion mutants competed either as well or better than the Lux+ strain at the root tip (Fig 3). Overall, the direction of fitness change as assessed in luminescence-based competition assays was consistent with the direction predicted by RB-TnSeq in 20 of 22 cases (91%, chi-squared *P* < 0.00012). Selected insertion mutants were further validated using an analogous LacZ blue/white screening approach, as well as through colonization assays with corresponding loss-of-function mutants generated by targeted mutagenesis (Materials and methods; S1 Data; S6 Fig). Although the magnitude of estimated fitness changes for individual genes varied across validation methods, the results were largely consistent with luciferase-based validation screens and confirmed in particular the impact of mutations in predicted colonization-depleted genes on colonization fitness (Materials and methods, S6 Fig, S1 Data).

We also explored dynamic aspects of root colonization, as the overall fitness of root colonizers might change over the days it takes to establish colonies on the root. To test whether fitness is static across a time course, we selected 4 predicted poor colonizers (*PS417_00160*, *PS417_01955*, *PS417_22145*, and *PS417_22775*) and 4 predicted enhanced colonizers (*PS417_08165*, *PS417_21035*, *PS417_03095*, and *PS417_10720*) to inoculate *Arabidopsis* seedlings in competition with the *LuxABCDE* expressing *P*. *simiae* strain as above but sampled at 1, 3, 5, and 7 days after inoculation. We measured the proportion of Luciferase-negative cells (i.e., mutant strain) from each root sample. Although all poor colonizers tended to grow more slowly once present on the roots, 1 (*PS417_22775*) failed to colonize very early on (S7 Fig). Most predicted enhanced colonizers appeared to grow more quickly than their poor colonizing counterparts, especially on later days (S7 Fig), although were still present in reduced numbers than expected, indicating a possible bias towards measuring luciferase-positive cells in this assay. Together, these detailed validation efforts support that the RB-TnSeq method applied to plant-bacteria interactions robustly defines both pronounced as well as subtle colonization defects.

### TnSeq across many in vitro conditions reveals functions of unannotated colonization genes

**Fig 4:**
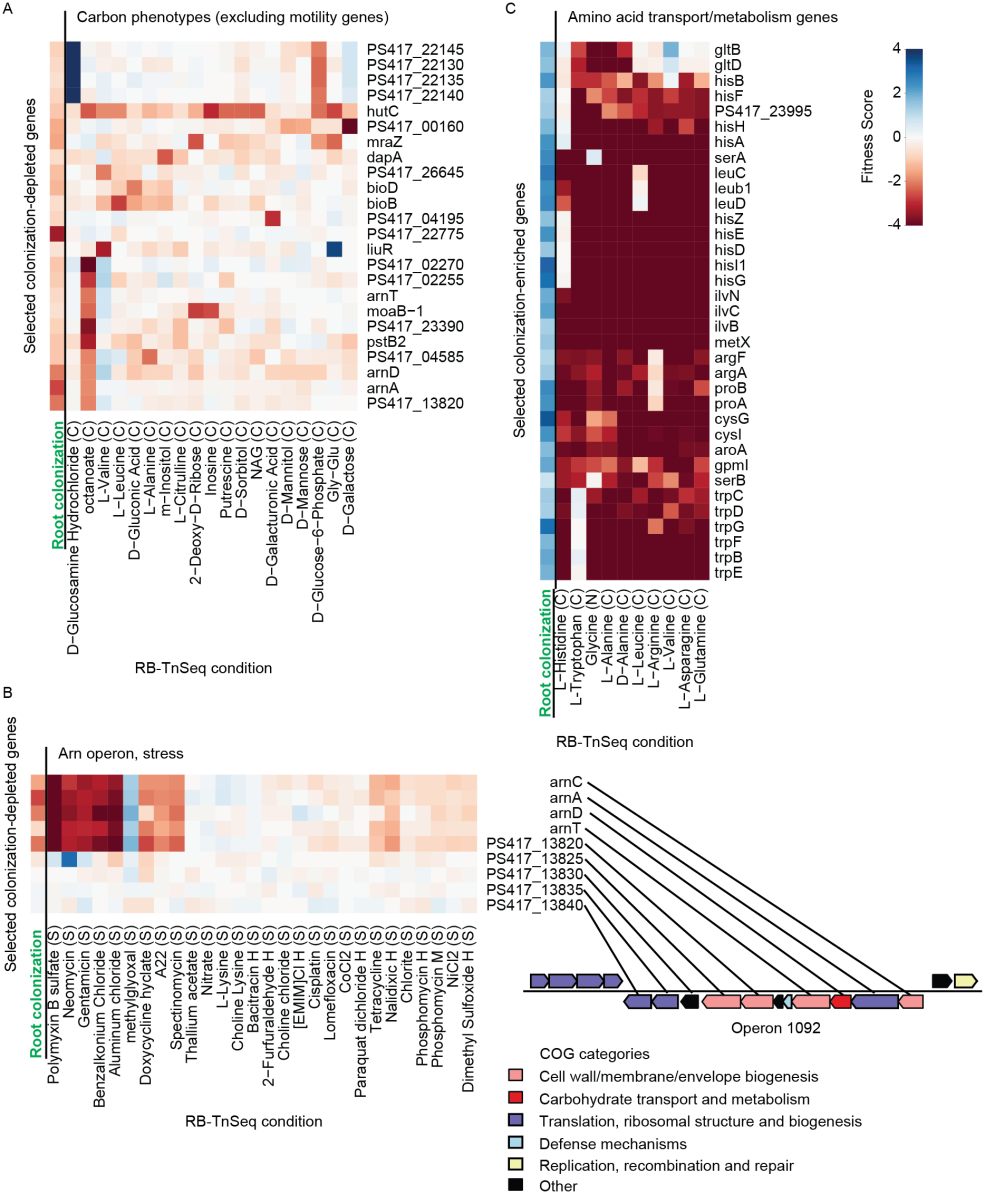
Selected groups of genes with reduced or enhanced fitness scores with putative functions highlighted by in vitro fitness data. Colonization-depleted (A and B) and colonization-enriched (C) genes were selected for their functional characteristics determined by in vitro growth assays. (A) Genes and in vitro conditions with at least 1 strong phenotype (|fitness score| > 2), excluding any gene with significantly reduced fitness in motility assays. (B) Operon (ID = 1,092) has 5 colonization-depleted genes, and is also required for resistance to antibiotics, including polymyxin B. (C) Colonization-enriched genes that also have significantly reduced fitness in many in vitro assays (see S8 Fig) with conditions in which the amino acids noted are the only carbon source. These genes have profiles consistent with amino acid auxotrophy. For (A), (B), and (C), gene names are presented on the right, data can be found in S1 Data. Conditions shown are labeled on the bottom. The color scale corresponding to the fitness score is shown at the top right. “Root colonization” (in green) refers to the root fitness score as described earlier (Results). Abbreviation: COG, cluster of orthologous group; RB-TnSeq, randomly barcoded transposon mutagenesis sequencing.

Many of the genes identified by our screen have no or at best vague annotations. To explore the physiological functions of the identified colonization genes in more detail, we compared our data to RB-TnSeq results of the same insertion mutant library tested under 90 distinct in vitro conditions, including 48 conditions using a defined compound as a sole carbon source in otherwise minimal media, 11 conditions using a defined nitrogen source, 29 stress conditions, and 2 in vitro motility conditions (inner and outer cuts of a soft agar motility assay) [23]. Although the complexity of individual phenotypes measured by these in vitro assays is considerably lower than that of root colonization processes, these assays are scalable and can thus be used to rapidly assess many metabolic or stress responsive functions. Within the large dataset covering genome-wide fitness across 90 conditions, we specifically examined the in vitro phenotypes of mutations in all 115 colonization-depleted and 243 colonization-enriched genes (Materials and methods, Fig 4 and S8–S10 Figs, S1 Data).

#### Colonization-depleted genes

Among the colonization-depleted genes, lack of motility (as measured by a soft-agar motility assay) featured prominently in the in vitro assays, as expected by their flagella-related annotations (S8 Fig). Beyond known motility genes, we also identified a hypothetical protein (*PS417_25790*) and an unannotated *OmpA* family protein (*PS417_05635*) as deficient in root colonization and motility (S1 Data). Although not unexpected due to the known importance of motility for colonization, this result confirms the general consistency between specific cellular functions revealed by in vitro RB-TnSeq experiments and in vivo RB-TnSeq assays. We also examined colonization-depleted genes regarding their importance for growth on specific carbon sources. We identified 24 genes (excluding any gene important for motility) with a strong growth phenotype on single carbon sources (Fig 4). Some of these genes (e.g. *PS417_00160*, *PS417_04195*, operon 1751 comprising genes *PS417_22130*–*22145*) appear to be involved in the metabolism of specific sugars (galactose, galacturonate, and glucose, respectively), suggesting a role for these carbohydrate sources in root-bacterial interactions. We also noted 2 genes (*PS417_08670* and *PS417_04595*) that, when mutated, caused slower growth in media with nucleosides (inosine or 2-deoxyribose) as the sole carbon source. Nucleosides were previously detected as components of root exudates of *Arabidopsis*, suggesting these microbes can use these compounds as an energy source [28]. Detailed in vitro profiling of mutants also provided insight into the biology of entire operons impacting colonization. For example, we observed an operon containing 5 consecutive genes with low fitness scores (operon ID 1,092, Table 1). Genes within this operon share significant homology to enzymes (*arnACDEFT*) that modify an arabinose residue within the lipid-A lipopolysaccharide cell wall component, previously shown to be important for evading host defenses [29,30]. Loss of function of these genes also confers susceptibility to lipid-binding polymyxin B stress (Fig 4) and several antibiotics (e.g., gentamicin, streptomycin), a trait known to be associated with colonization efficiency in other colonization paradigms[12,31,32]. However, in contrast to these previous reports, operon ID 1,092 in WCS417r appears to be distinct in its mode of action, consistent with the existence of an alternative pathway to evade lipopolysaccharide-binding antimicrobials.

#### Colonization-enriched genes

Among the colonization-enriched genes, we observed many that confer strongly reduced in vitro fitness in a broad range of single carbon or nitrogen source assays when disrupted (Fig 4 and S8 Fig). These genes are often annotated as amino acid transport or metabolism components. *Arabidopsis* root exudates are known to contain various amino acids [28], suggesting that the apparent amino acid auxotrophy of these mutants confers enhanced fitness on the root relative to the comparatively nutrient-poor support medium. Thus, in the absence of these stress conditions, it appears plausible that such mutants have increased fitness compared to the WT strain due to the metabolic expense associated with the expression of amino acid transport/metabolism pathways. Apart from genes involved in amino acid metabolism, we also observed several colonization-enriched genes colocalizing within operons. For example, mutations in all genes within operon 228 confer enhanced colonization ability. Operon 228 contains genes with homology to genes known to be required for *Flp* Type IV pilus assembly (*CpaBDEF* and *TadBC*). Although previous studies suggested these genes are needed for root colonization [12], we note that the host previously described (tomato) may be a different enough niche from *Arabidopsis* roots to require different attachment or motility mechanisms for successful colonization. We also speculate that mutations in this pilus locus may promote a more planktonic lifestyle in colonizing bacteria, resulting in decreased cell–cell and cell–surface interaction, and increased motility and colonization efficiency. Indeed, a different study demonstrated that reduction in biofilm formation mediated by mutations in *GacA*/*GacS* resulted in a hypermotile phenotype leading to increased *P*. *fluorescens* colonization of roots compared to the WT strain [33]. Another study suggested that Type IV pili inhibit sliding motility in situations where the flagella are not abundant [34], which might be relevant to the plant root environment, as recognition of flagellar proteins can trigger the plant immune system [35]. Taken together, integration of our colonization phenotypic data with in vitro metabolic, motility, and stress data reveals specific cellular and molecular processes involved in root colonization and offers clear hypotheses for the investigation of these phenotypes.

#### Under-annotated genes

The unbiased, genome-wide profiling of RB-TnSeq libraries across a wide range of in vitro conditions is particularly useful to obtain initial insights into the function of genes otherwise lacking useful annotations. Our colonization assays identified 44 hypothetical or otherwise underannotated colonization genes, including 21 hypothetical proteins (8 depleted, 13 enriched). COG categories represented among these 44 genes included 12 genes with “unknown function” (4 depleted, 8 enriched), 15 genes with “general function prediction” (2 depleted, 13 enriched), 1 with “cell wall/membrane/envelope biogenesis” (depleted) but with a “hypothetical protein” functional prediction, and 16 genes without COG annotations (S1 Data). In vitro fitness assays showed that more than half of the underannotated genes (27 of 44, 61%) have significantly altered fitness under at least 1 condition (S10 Fig). Four of these genes appear to be necessary for growth under a set of single-carbon source conditions, 9 show strong but diverging phenotypes under several stress conditions (i.e., mutant strains for these genes grow much faster under some stress conditions, but slower in others). Other genes appear to be important under very specific conditions, such as growth in inositol or valine as a sole carbon source (S10 Fig). Intriguingly, 17 mutants (39%) in under-annotated loci did not show significant phenotypes in any of the in vitro assays, suggesting that the corresponding genes are involved in functions incompletely captured by in vitro conditions and highlighting the utility of RB-TnSeq screens for studying complex processes such as root colonization.

## Discussion

We developed a genome-wide map of microbial genes required for colonization of plant roots in a plant/microbial system. Building on the successful application of RB-TnSeq for the large-scale assessment of in vitro phenotypes [23], the present study demonstrates the utility of this experimental paradigm for studies of bacterial plant root colonization in vivo, thus applying it to a process that considerably exceeds in vitro assays in terms of complexity. By using the colonization of *Arabidopsis* roots by the biocontrol bacterium, *P*. *simiae* WCS417r, as a model of colonization, we observed a substantial variety of genes conferring altered survivability to the bacteria when mutated, mirroring the complex nature of this interaction system. One challenge of TnSeq assays in general, and TnSeq assays targeting colonization phenotypes in particular, is the reliance on a diverse population of insertion mutants in the colonized host after coincubation. During *Arabidopsis* colonization by *P*. *simiae*, we found that only 100 to 1,000 independent colonization events occur per individual root, creating a potential bottleneck for downstream analysis. We mitigated this effect by sampling large numbers of plants (approximately 1,000 pooled seedlings per sample), resulting in the recovery of most constituent mutant strain barcodes in every pooled sample. Additional confounding factors include environmental or community considerations, namely that survival of individual mutants on the plant support medium and filter prior to root colonization might be reduced, which need to be corrected with appropriate controls (S3 Fig, Materials and methods). Certain functional deficiencies, especially those associated with secreted or extracellular activity, of some colonization genes might be effectively rescued by a largely WT population for that function. Furthermore, some mutants identified by RB-TnSeq showed quantitatively weaker or no significant phenotypes in validation screens, raising the possibility that their fitness is higher when they are rare members of a diverse mutant population, as opposed to validation experiments where these mutants represent 50% of the population. Notwithstanding these limitations, the high-validation rate of colonization genes in secondary validation assays supports the robustness of our genome-wide map of root colonization.

Many genes with significant fitness scores clustered within operons, further reinforcing the validity of RB-TnSeq-derived results. Indeed, colonization genes within the operons shown in Table 1 represent the majority of genes included within these operons. Additionally, 98 colonization genes were not predicted to be part of an operon or were part of an operon of only 2 to 3 genes. Some colonization genes occurred in operons in which only 1 or a small subset of genes showed significant fitness scores. For these operons, it is possible that not every gene is required for the given function, or that individual enzymes are shared across alternative pathways. These results, along with the observation that many of the genes identified from our screen are involved in processes well known to be vital to colonization of plants (e.g., motility, carbohydrate utilization) are consistent with the notion that fitness scores from genome-wide colonization reflect valid, biologically relevant genes and pathways.

We also compared the list of genes significantly affecting colonization to known colonization genes based on a number of smaller-scale mutant screens in *P*. *putida* [21] and found that 20 out of 87 *P*. *putida* homologues with colonization data in our screen showed altered fitness (S1 Data). Although this limited overlap is expected due to the heterogeneous nature of the assays performed across multiple studies in *P*. *putida*, as well as known differences between organisms and hosts, they further strengthen the biological validity of the genome-wide colonization map generated in the present study.

We observed a surprisingly large number of genes with positive colonization fitness scores (243 positive versus 115 negative). While most of these mutants showed quantitatively less pronounced phenotypes in luciferase-based screens than predicted by the initial RB-TnSeq scores, in almost all cases the direction of the effect was confirmed (Fig 3). This observation, along with the propensity of colonization-enriched genes to colocate in operons, supports the conclusion that the predicted phenotypes for these genes are biologically relevant. A large proportion of these genes encode proteins involved in amino acid transport and metabolism (Figs 2 and 4), suggesting that auxotrophy for certain amino acids confers a selective advantage for survival in the plant-associated environment rich with exuded amino acids and sugars. Two of these genes (*PS417_01565* and *PS417_21035*) were assayed in our luciferase-based competitive colonization screen, and behaved as predicted by the RB-TnSeq data. This poses an intriguing technological opportunity: engineering strains to be more dependent on their plant hosts may have the dual effect of improving colonization while simultaneously restricting the survivability of engineered strains outside the context of the root.

Lastly, we identified 44 genes that had vague or no annotation information. A particularly noteworthy subset of these genes lie within operons with multiple mutants with colonization phenotypes, yet without clear functional annotation. These may represent truly novel genes or pathways contributing to colonization of and survival on roots and the results from our in vivo and in vitro screens pave the way for their targeted functional and biochemical characterization. In summary, the genome-wide map of plant colonization genes described in the present study highlights diverse metabolic and physiological functions that support or hinder plant-microbe association and points to novel functions mediating this process.

## Materials and methods

### Plant growth conditions

*A*. *thaliana* Col-0 seeds were surface-sterilized in 70% ethanol for 5 minutes, followed by 10% bleach plus 0.1% Triton-X100 for an additional 5 to 10 minutes. Sterilized seeds were washed 5 times in sterile water, and stratified in the dark for 2 to 3 days at 4°C. After stratification, 100 seeds were plated on a nylon mesh filter (100 micron pore size, cut to an area of approximately 8 cm^2^ [B0043D1XRE Amazon.com Inc, Seattle, WA]) placed on top of plant growth media (0.5X Murashige and Skoog basal salts [MSP01, Caisson Laboratories, Smithfield, UT], 2.5 mM MES [M3671, Sigma-Aldrich, St. Louis, MO], 0.6% phytagel [P8169, Sigma-Aldrich], pH 5.7) in a 10 cm square petri dish. Seedlings were grown upright in a Percival incubator (CU-36L5, Geneva Scientific, Williams Bay, WI) for 7 days prior to treatment.

### Bacterial strains, growth, and inoculation

A cultured isolate of *P*. *simiae* (WCS417r) was obtained from Dr. Corné Pieterse (Utrecht University). The barcoded insertion library for this strain was generated by transposon mutagenesis with a barcoded mariner transposon library, followed by TnSeq mapping and barcode association, as previously described [23,24]. Glycerol stocks of this library were used for subsequent experiments, stored in 1 mL aliquots containing approximately 4 x 10^8 cells/mL. On average, this represents greater than 1,000-fold excess of each individual strain and should avoid any filtration or passage effects associated with recovery from glycerol stocks. A *LuxABCDE*-expressing transformant (WCS417r:Lux+) was generated by inserting an IPTG-inducible expression cassette using a *mariner* transposase system, such that Luciferase expression could be visualized following IPTG induction. The insertion site of the *LuxABCDE* transgene was determined to be at approximately position 1628942. WCS417r, WCS417r:Lux+ cultures were grown in LB Lennox media at 28°C in a shaking incubator at 200 rpm. The insertion mutant library and single insertion mutants were grown in LB Lennox supplemented with 100 μg/mL kanamycin at 28°C in a shaking incubator at 200 rpm. LacZ-expressing WCS417r was created using a previously engineered miniCTX-lacZ vector driven by the *Vibrio cholera lacZ* promoter[36]. Briefly, *lacZ* was transferred to the neutral phage attachment site (attB) of WCS417r via biparental mating using *Escherichia coli* SM10 and selected on LB plates containing 75 μg/mL tetracycline.

### Isolation of mutant strains from colonization experiment

Each colonization experiment was comprised of 5 replicates of each sample type. Three colonization experiments were performed at the DOE Joint Genome Institute (sets A, B, and C). For a single colonization experiment, a glycerol stock containing the transposon library was inoculated in 50 mL fresh LB and grown for approximately 6 hours until the culture reached the midlog phase (OD between 0.2 and 0.6). Cells were then harvested by centrifugation (3,000 g for 3 minutes) and washed 3 times by resuspending in 1 mL of 0.5X MS media and pelleting the cells. After washing, the cells were resuspended in 1 mL, 0.5X MS, and the OD of the resuspension was calculated by spectrophotometer (using a 1:10 dilution). Cells were then normalized to OD 0.5, and 50 μl (corresponding to approximately 1.0 x 10^7 cells) was spread onto 0.5X MS phytagel (0.6%) plates using sterile glass beads. Seven-day-old *Arabidopsis* seedlings grown on a sterile nylon mesh filter (110 μm pore size) laid on top of 0.5X MS phytagel (0.6%) plates were transferred by lifting and replacing the filter onto the inoculated plates. Five aliquots of the OD 0.5 culture were saved as an input (IPT) culture for each experimental replicate (set). Ten plates were inoculated with bacteria and exposed to a nylon mesh filter without seedlings. Five such filters (NRI) were allowed to contact the bacteria plate for 1 hour before being used to inoculate 50 mL LB + Kanamycin (100 μg/L) overnight. The remaining 5 filters (NRF) along with the plate/filters containing Col-0 seedlings were incubated vertically in a Percival growth chamber for 7 days under short-day (8 hour light/16 hour dark) conditions (22°C). Following cocultivation, the NRF filters were used to inoculate a 50 mL LB Lennox + Kanamycin (100 μg/L) culture, and grown overnight. Seedlings on plates containing bacteria were then cut just below the root/shoot junction, and the isolated roots were placed into 10 mL, 0.5X MS liquid. Ten plates of roots were pooled into a single sample. The pooled roots were vortexed for 15 seconds to wash loosely adhered cells from the surface of the roots, and the buffer removed. The washing procedure was repeated 5 more times (total 6 washes). The washed roots were then cut into thirds, placed into 2 mL eppendorf tubes with 2 metal beads and 200 μl, 0.5X MS liquid. The roots were ground in a TissueLyser bead mill for 2 cycles of 5-minute grinds at 30 Hz (Qiagen, Hilden, Germany), inverting the tubes between cycles. Ground roots (rhizoplane + endophytic compartment; RPL) were used to inoculate 50 mL LB Lennox + Kanamycin (100 μg/L) cultures overnight. Two mL samples from all overnight cultures (IPT, NRI, NRF, RPL) were harvested after 12 to 16 hours of growth, pelleted, and stored at −80°C prior to DNA extraction.

### DNA isolation and library preparation

DNA from frozen pellets was isolated using the DNeasy Blood and Tissue kit (Qiagen, Hilden, Germany) according to the manufacturer’s instructions. DNA was quantified with a Qubit fluorometer (Thermo Scientific, Raleigh, NC) according to the manufacturer’s instructions and normalized to 10 ng/μl. Samples with (DNA) less than 10 ng/μl were not diluted. Twenty microliters of the normalized (or undiluted, in the case of low concentration samples) DNA was used as template in a PCR using primers flanking the transposon barcode region, each containing an Illumina adapter and multiplexing index sequence (BarSeq) [23,24]. PCR was performed using Q5 DNA polymerase with Q5 GC enhancer (New England Biolabs, Ipswich, MA) for 25 cycles of 30 seconds at 98°C, 30 seconds at 55°C, and 30 seconds at 72°C, followed by a final extension at 72°C for 5 minutes. Following PCR, 10 μl of each reaction was pooled into 3 sets of 25 (sets A, B, and C; see S1 Data) amplicon libraries, corresponding to each experimental set (see previous section). Three pooled libraries (representing sets A, B, and C) were then purified using the DNA Clean & Concentrate Kit (Zymo, Irvine, CA) according to the manufacturer’s instructions. Each set was sequenced on its own lane on an Illumina HiSeq 2500 machine using the 1T paired-end, 2 x 101 cycle protocol, producing an average of 3 to 8 million reads per sample.

### Identification of putative colonization genes

We used barcode sequencing to quantify the representation of mutants in each sample and compared barcode frequencies across samples [24]. Raw sequence reads were initially processed by looking for the 6 nt adapter sequences on either side of a 20 nt random barcode. Reads with exactly 20 nt barcode sequences, no mismatches between mate-pair barcodes, and high-quality scores (Q > 30) from each of the 60 libraries (15 IPT, 15 NRI, 15 NRF, and 15 RPL) were then saved into filtered fastq files and used as input into the BarSeqR pipeline [24]. For this analysis, the NRI samples were set as “Time0” controls, with all samples normalized to these samples. The NRI samples were used as the normalization controls to factor out any amplification effects caused by overnight culturing. To assess the saturation of our sampling method, we quantified the number of barcodes (and genes mutated) in all 60 samples. On average, for each sample, we recovered >80% of the insertion mutants that we had mapping information for. We also quantified the number of barcodes and genes with mutations when samples were considered together (combining the unique barcode and gene count represented by any of the samples within a given sample type, e.g., NRF or RPL). With replication, our recovery rates approach saturation for each of the 4 sample types (S11 Fig). The BarSeqR scripts report per-gene fitness scores (normalized log-ratio) and *t*-like test statistics indicating the relative effect size and significance between each sample and the average of the Time0 controls, respectively, for each of the 60 samples, including the Time0 controls (NRI samples) [24]. After normalization of total counts across samples, we determined 3 separate derived fitness scores for each gene. Each of these scores measures a different potential effect influencing microbial growth in these experimental conditions: A mesh fitness score comparing the NRF and NRI samples and thus measuring changes in the ability to growth on the nylon mesh alone; a root + mesh fitness score comparing the RPL and the NRI samples, which measures the overall ability to grow on the root and the nylon mesh; and a root fitness score, comparing the RPL and NRF samples directly, which represents the root + mesh fitness score corrected for the mesh fitness score to quantify the ability to grow on the root after correction for mesh-related effects.

To classify genes based on their mesh phenotype, we compared fitness scores from the various sample types and computed 3 derived fitness scores, looking for significant differences based on an empirical *P* value corresponding to an FDR of 0.05 (Student *t* test) and an effect size (absolute difference between the means) of > 0.5: a root + mesh fitness score (comparing RPL to NRI; *P* < 0.014), a mesh fitness score (comparing NRF to NRI; *P* < 0.01) and a root fitness score (comparing RPL to NRF; *P* < 0.013). Considering that weak colonization fitness scores may not be biologically meaningful, we chose a threshold effect size cutoff of 0.5, which eliminated nearly half of the genes that were significant based on *P* value alone. We binned these genes into 2 main groups (S3 Fig). Genes in group 1 (gray, S3 Fig; 149 genes) had significant root + mesh fitness scores, but the quantitative magnitude of this effect was largely explained by changes to the ability to survive on the nylon mesh alone (mesh fitness score). Consequently, genes in group 1 were considered low-confidence candidate genes for root colonization, despite their significant root fitness scores. Genes in group 2 (blue and cyan, S3 Fig; 358 genes) exhibited significant root fitness scores of at least moderate effect sizes (root fitness score absolute value >0.5). A subset of these (group 2b cyan, S3 Fig; 75 genes) additionally did not exhibit significant root + mesh fitness scores”. Mutations in these genes likely confer altered fitness on mesh, but this phenotype was at least partially compensated for by the presence of roots. Despite this more complicated phenotype, we included these genes in group 2 because they are likely to be involved in root colonization.

### Homology-based search for predicted function or conserved domains (BLAST)

Putative essential genes in the *P*. *simiae* genome were identified by a BLAST homology search using as a query protein sequences of *P*. *simiae* genes, and as a subject database genes characterized as essential from representative gamma-proteobacteria within the Database of Essential Genes [37]. Alignments between *P*. *simiae* and DEG protein sequences having more than 50% identity across at least 80% of both subject and query sequences, and having an e-value <1e-50 were considered to be significant, and those *P*. *simiae* proteins were classified as homologous to an essential gene.

### RB-TnSeq in vitro growth assays

For RB-TnSeq growth assays in defined media, we incorporated the *P*. *simiae* WCS417r strain into a larger microbial functional genomics effort to be published soon [23]. As part of this effort, growth assays with carbon sources, nitrogen sources, and inhibitors and soft agar motility assays were performed as previously described [23,24].

For comparing colonization to in vitro assays, we used a simplistic threshold to assign a phenotype”of abs (in vitro assay fitness score) >1, and a strong phenotype of abs (in vitro assay fitness score) >2, as described [24].

### Arraying and selecting clones from the library

An aliquot of the RB-TnSeq library was grown to an optical density of approximately 0.2, diluted approximately 1 to 50,000, and plated on LB + kanamycin agar plates. These plates were incubated for 64 hours at 18°C, with limited lighting until single colonies were apparent. Colonies were then selected by a Qpix460 (Molecular Devices, Sunnyvale, CA) and arrayed into 384-well plates (64 plates total) containing 90 μL LB with 7.5% glycerol and kanamycin and were grown overnight in the dark at 29°C until they reached an OD450 of 0.5. Aliquots were taken for downstream processes and the 384-well plate was kept at −80°C. To unambiguously determine the barcode identity and plate address of all the mutants in this collection, 25 nL from each well of the 384-well plates was transferred using an Echo525 instrument (Labcyte, San Jose, CA) to a new well on a 96-well plate based on a multiplexing strategy involving pooling of rows, columns, and plates. 1.6–4.8 μl of each pooled culture within the 96-well plate was used as input for RB-TnSeq. Following sequencing of the 96 RB-TnSeq libraries on a MiSeq v2 instrument, an in-house script for determining the address for each barcode within the clone library (based on the pooling schematic) was used. To isolate specific barcoded insertion mutants, the 384-well glycerol stock plate containing the clone of interest was removed from −80°C and kept on dry ice. A sterile loop was used to scrape the surface of each frozen stock well and streak LB+kanamycin agar plates. Following an overnight incubation at 28°C, 3 to 6 colonies were individually picked and grown in 100 μl LB with Kanamycin for 6 to 8 hours at 28°C. An aliquot of this culture was diluted 1:10 in water, and used as input for qPCR (40 cycles) using primers flanking the barcode region. Positive qPCR wells were cleaned with ExoSAP-IT and sent for Sanger Sequencing to confirm the correct barcode sequence. Cultures started from individually-picked colonies that were confirmed with Sanger data were then used for phenotypic analysis in planta.

### Luciferase-based competitive colonization assay

To independently test whether isolated mutants had altered colonization ability, we designed a bioluminescence assay using the Lux+ strain described above. When mixed at a defined ratio with individual nonluminescent mutant strains, this Lux+ strain allows for a direct, luminescence-based in planta quantification of competitive colonization, in which luminescence intensity inversely correlates with the proportion of the unlabeled strain on the root (Fig 3 and S5 Fig). This ratio gradient can be used to derive a standard curve with which we can normalize root colonization measurements (by luminescence) with mutant strains. In this assay, 24 bacterial cultures, including the 22 insertion mutant strains, the parent WCS417r strain, and the engineered Lux+ strain, were grown overnight, and subcultured the following morning into 5 mL LB (+ Kan for the insertion mutant strains). After reaching an OD of between 0.2 and 0.6, the cultures were pelleted at 3,000 g, and washed 3 times with 1 mL, 0.5X MS. After the third wash, each culture was resuspended in 1 mL, 0.5X MS, and measured for optical density. Each culture was then normalized to an OD of 0.5, and 30 mixtures were made: a WT/Lux+ standard curve series (100% WT/0% Lux+, 80% WT/20% Lux+, 60% WT/40% Lux+, 50%WT/50% Lux+, 40% WT/60% Lux+, 20%WT/80% Lux+, and 0%WT/100% Lux+), a buffer control mixture (50% Lux+, 50% 0.5X MS), and 22 mixtures of 50%Lux+/50% insertion mutant strain. 50 μL of each mixture was then spread onto 2 sets of separate 0.5XMS (100 μm IPTG, 0.6% phytagel) plates (60 plates total) using sterile glass beads. Squares of sterile nylon mesh were placed on one set of 30 plates, while 7-day-old *Arabidopsis* seedlings grown on nylon mesh were transferred to the other set of 30 plates. All 60 plates were sealed with micropore tape and incubated vertically in a short-day light chamber for 7 days as for the initial colonization screen.

On day 7, the plates containing only nylon mesh (and bacteria) were opened, and the filter was vortexed in a 15 mL tube with 5 mL, 0.5X MS for 10 seconds. An aliquot was sampled from this mixture, and 5 serial dilutions were prepared. Twenty microliters from each dilution were spotted into single a cell of a 6 x 6 gridded LB agar plate and incubated overnight (to estimate the ratio of the luciferase strain to the WT luciferase strain on mesh alone). Each seedling plate was then removed from the growth chamber, and 5 seedlings from each plate were placed onto a large 0.5X MS plate (100 μM IPTG, 0.6% phytagel) and imaged using an epi-white illumination for a 2 second exposure and in complete darkness for a separate 30 minute exposure using a GelDoc gel imager (Bio-Rad, Hercules, CA). The following day, the colonies from the LB plate spotted with the mesh-derived dilution series were imaged in a GelDoc imaging platform, taking both transblue images (approximately 0.01 second exposure) and dark (no illumination) exposures.

Bioluminescence from each seedling imaged was quantified by determining the integrated pixel density within a 10 x 30 pixel rectangle surrounding the basal region of the root, with the lower boundary of each rectangle positioned just above the root tip, and then subtracting from this value the integrated density of a 10 x 30 pixel rectangle just below the root (Fig 3) without overlapping the root region. The resulting values from each of the 5 seedlings per sample type (e.g., WT/Lux+ standard curve or mutant/Lux+ mixture) were then used to determine the mean root intensity for that sample. The estimated root ratio of mutant/Lux+ was determined by normalizing the root intensity for each of the mutant/Lux+ roots to a linear regression model of the WT/Lux+ ratio series. To determine the ratio of each unlabeled (i.e., WT and mutant strains) to the Lux+ strain in all the mixtures derived from the mesh filter alone, colonies were counted from dilutions where typically between 30 and 300 colonies were visible on a single cell in the 6 x 6 gridded plate. These values (mesh ratios) are expressed as the ratio of unlabeled colonies to the total number of colonies. A least-squares linear regression was then computed from the mesh ratios and root intensities of the WT/Lux+ series. The slope and intercept of this regression was used to determine the estimated ratio of unlabeled strains to Lux+ strains on the root. The colonization index was defined as the natural log of the estimated root ratio minus the natural log of the observed mesh ratio. This procedure was performed independently 3 times.

### Time course analysis of selected insertion mutant strains

Eight strains were selected for additional validation experiments over a 7-day time course: 4 with insertions in predicted depleted colonization genes (*PS417_00160*, *PS417_01955*, *PS417_22145*, and *PS417_22775*) and 4 with insertions in predicted enhanced colonization genes (*PS417_08165*, *PS417_21035*, *PS417_03095*, and *PS417_10720*). Briefly, 5 mL cultures of each strain, the WT strain, and the Lux+ strain were grown individually in LB Lennox overnight to midlog phase, and then pelleted, washed 3 times, and normalized to OD 0.5 in 0.5X MS media. Sixteen populations were then created: 8 1:1 mixtures of the Lux+ strain with the mutant strains (1 mixture each), and 8 mixtures of WT and Lux+ strains at different ratios (100%/0%, 85%/15%, 70%/30%, 50%/50%, 40%/60%, 25%/75%, 10%/90% and 0%/100% WT/Lux+ strains). Each population was inoculated onto 2 separate, 0.5X MS/0.6% phytagel plates (50 μl used for each inoculation), totaling 32 plates for each experiment. Four squares of nylon mesh (approximately 3 cm^2^ each) were added to each of 16 out of the 32 plates. Nylon mesh (approximately 8 cm^2^ each) supporting approximately one hundred 7-day-old *Arabidopsis* seedlings were transferred to the remaining 16 plates. After 1, 3, 5, and 7 days following inoculation, 1 nylon mesh square from each mesh-containing plate was vortexed in 1 mL 0.5X MS media for 30 seconds, and then placed onto a large square petri dish containing 0.5X MS and 0.6% phytagel and 10 μm IPTG (to induce luciferase expression), leaving behind 1 mL of the mesh population. Additionally, 3 seedlings from each of the plant-containing plates were transferred to same large plate and allowed to incubate at room temperature for 2 hours prior to imaging as described in the previous section. Root length and mesh area were measured from each image, along with total luciferase intensity (though this was not a reliable metric especially on days 1 and 3 when the intensity was very dim). Following imaging, the 3 selected roots were excised, placed into 300 μl 0.5X MS in 1.5 mL tubes, sonicated at low power (160W at 20 kHz for 30 seconds), and vortexed for 30 seconds at high speed, resulting in the root population. 20 μl of the root and mesh populations were diluted separately (for each mutant and WT/Lux ratio mixture) into 6 serial 10-fold dilutions, plated (15 μl each) onto LB Lennox and 10 μm IPTG and 1.5% agar plates, and incubated for 24 hours. Each plate was then imaged with transblue illumination and with no illumination (60 second exposure) to visualize all colonies and Lux+ colonies. Both images were overlaid, and Lux+ and Lux- CFUs were counted for dilutions in which single colonies could be visualized. Root CFUs (Lux+ and Lux-) were used to compute the competitive colonization ability for each mutant, while the mesh CFUs and total CFUs from the root sample were used to compute the overall population sizes per unit length (for roots) or area (for mesh).

### Generation of *P*. *simiae* deletion mutants for experimental validation

Deletion mutation alleles for *PS417_08425*, *PS417_19755*, *PS417_08190* were constructed using splice-overlap extension PCR and WCS417r genomic DNA as a template. Deletion alleles were cloned into suicide vector pDONRX via Gateway cloning [38]. Resultant plasmids were confirmed by Sanger sequencing and introduced into WCS417r via biparental mating with SM10. Unmarked double-crossover mutants were then isolated using sucrose-mediated counter-selection as previously described [39] and confirmed by PCR.

### LacZ competition assays

WCS417*r*:*LacZ+* and either unmarked WCS417r or mutant derivatives were mixed 1:1, as determined by OD_600_, before 2 x 10^4^ CFU were applied to the surface of 0.5X Murashige and Skoog plates containing 0.6% phytagel. Approximately fifty 1-week-old *Arabidopsis* seedlings germinated on nylon mesh were transferred to phytagel plates containing bacterial mixes and allowed to coincubate for 24 hours. Roots were separated from shoots, and washed 3 to 4 times prior to lysis. LacZ positive colonies were enumerated by plating serial dilutions of root homogenates and nylon mesh washes on LB media containing 40 μg/mL X-gal. Competitive indices were computed based on the white:blue ratio. Although the results from assaying many of the mutant strains under the LacZ and Lux+ assays agree, there was variability between assays. This was largely expected, given that the validation assays have higher degrees of variability compared to the RB-TnSeq screen, and the scale of the data is vastly different (tens of colonies per replicate for the validation assay versus hundreds to thousands of counts for the RB-TnSeq screen).

## Supporting information

S1 Fig Properties of insertion mutant library. Mariner mutagenesis of P. simiae WCS417r and subsequent TnSeq resulted in a library of 110,142 individual mutant strains with mapped insertion sites and unique barcodes, distributed across the genome at an average rate of 18 insertions per 1000 bp (A). Of the 5,610 genes in the genome, we generated mutations in 4,709, with most genes having fewer than 50 insertions (B). The number of insertions found within a gene is largely a function of the number of thymine-adenine (TA) dinucleotide sites (C). 385 out of the 827 genes with no insertion mutants, and 307 of the 4,783 genes with viable insertion mutant strains represented in the library share significant homology with genes in the Database of Essential Genes (C, black points). TnSeq, transposon mutagenesis sequencing.
(TIF)

S2 Fig Overview of colonization screen methods. Bacterial samples used for sequence-based identification of colonization genes were collected by inoculating the RB-TnSeq insertion mutant library on nylon mesh supporting Col-0 seedlings, then culturing the colonized roots (excised and washed) in rich media overnight. Control samples also collected for this analysis included an input library control (collected from the inoculation culture), as well as overnight cultures of colonized nylon mesh without plants incubated for 1 hour or 7 days. RB-TnSeq, randomly barcoded transposon mutagenesis sequencing.
(TIF)

S3 Fig Genes contributing to fitness on roots compared to a mesh filter. Fitness scores of root-derived mutant strains (normalized to the fitness scores derived from pre-colonization NRI samples) are plotted (y-axis) against fitness scores from post-colonization NRF-derived mutant strains (also normalized to the NRI mesh input samples; x-axis). Points colored in gray correspond to genes that are significantly enriched or depleted between “Root” (RPL) and NRI samples, but not significantly different between RPL and NRF samples (group 1). Points colored in cyan represent genes significantly different between RPL and NRF samples, but not significantly different between RPL and NRI samples (group 2a). Points colored in dark blue correspond to genes significantly different between RPL and NRF samples, as well as between RPL and NRI samples (P < 0.01, effect size > 0.5) (group 2b). The blue- and cyan-colored genes are considered to be significant colonization genes. Genes circled in red are the 22 genes selected for further validation. NRF, no root final; NRI, no root initial.
(TIF)

S4 Fig Distribution of fitness scores, significance thresholds. To determine which genes significantly contribute to root colonization, we performed a two-stage thresholding strategy. Genes having P values < 0.013 (t-test; FDR = 0.05; dashed line; genes above shown in blue and green) and having an effect size (absolute value of the fitness score) > 0.5 (dotted line, genes above or below shown in red and green) were determined to be significant (shown in green). Across the 4,576 genes considered in this screen, most fitness values were normally distributed about zero, although the fitness score distribution contained a relatively long high-fitness value tail (upper right quadrant). FDR, false discovery rate.
(TIF)

S5 Fig Standard curve generation for Lux+ assays. Root intensities of independent competition experiments are plotted against observed WT and Lux+ ratios obtained from inoculated phytagel plates/mesh without roots. Standard curves for three independent assays (corresponding to the three independent biological replicates performed for each insertion mutant strain) are shown. See S1 Data for numerical values. Lux+, luciferase producing; WT, wild-type.
(TIF)

S6 Fig Multiple assays used to validate selected insertion mutant strains. LacZ- and a Lux+ based methods for quantifying competitive colonization ability were performed on a set of insertion mutant strains (S1 Data, Materials and methods). The Lux+ method was performed on all 22 insertion mutant strains (green), the LacZ method was performed on 12 of these 22 mutant strains (orange). In addition, 4 genes were mutated via targeted deletion, and were also assayed using the LacZ method (black). Plotted are the colonization indexes for each assay (y-axis) against the Root fitness score determined by the RB-TnSeq method (Materials and methods). Error bars represent the standard error of the mean (n > = 3 replicates for y-axis, n = 15 for x-axis). Lux+, luciferase producing; RB-TnSeq, randomly barcoded transposon mutagenesis sequencing.
(TIF)

S7 Fig Time course analysis of competitive colonization. We selected eight mutants and subjected them to a competitive colonization assay with the LuxABCDE expressing P. simiae WCS417r strain as described previously (Fig 3), though harvesting cells for quantification using a sonicating water bath followed by CFU counting for to estimate the ratio of Lux+—and to Lux- + cells from roots following 1, 3, 5, or 7 days after initial inoculation (see Materials and methods). Log 10 total cells recovered from 3 sonicated roots are shown in blue, and the estimated number of cells that are Lux- (i.e. colonization mutant) are shown in red. (A) The fraction of mutant cells in each root population are shown in red (for predicted colonization depleted mutants) and blue (for predicted colonization enriched mutants). Error bars represent the standard error of the mean (n = 3 independent replicates). We also measured total cell counts for each population grown on nylon mesh alone (B) or roots (C) over the same time course, noting the slow decline of bacteria grown on mesh versus the exponential growth of cells grown on roots. See S1 Data for numerical values. CFU, colony forming units; Lux, luciferase.
(TIF)

S8 Fig Fitness of putative colonization genes in various in vitro-defined conditions. Heatmaps of in vitro fitness data for all colonization genes. All data was clustered row- and column-wise using an unsupervised hierarchical clustering algorithm (R hclust package), re-ordered to separate colonization-depleted and enriched genes (preserving the clustering order within each category), and then split to separate single carbon/nitrogen sources (A) and motility or stress conditions (B). Color scale is shown at the bottom right. Data used to generate these heat maps is included in S1 Data.
(TIF)

S9 Fig In vitro phenotypes for selected validated insertion mutant strains. 22 insertion mutants were selected based on their predicted function or the severity of their defect in colonizing the root. Heatmaps (unclustered) of in vitro and root fitness data for these 22 colonization genes are split to separate single carbon/nitrogen sources (A) and motility or stress conditions (B). Color scale is shown at the bottom right. Data used to generate these heat maps is included in S1 Data.
(TIF)

S10 Fig In vitro phenotypes for under-annotated genes. 44 Under-annotated genes with significant root colonization fitness scores were selected based on their COG category (No COG annotation, General Function Prediction Only, Unknown Function) or gene description (hypothetical protein). In vitro and root fitness scores for all conditions are shown here. The data was clustered row- and column-wise using an unsupervised hierarchical clustering algorithm (R hclust package) and then split to separate genes with a phenotype (|fitness score| > 1) in any one in vitro condition (left) or genes with no apparent phenotype under any in vitro condition (right). Color scale is shown at the top left. Data used to generate these heat maps is included in S1 Data. COG, cluster of orthologous group.
(TIF)

S11 Fig Barcode and gene insertion recovery metrics. Barcode and gene insertion recovery metrics. Number of (A) unique genic barcodes (associated with insertion sites between 10% and 90% of a gene body) recovered and (B) gene knockouts represented from 5 replicates of each sample type, separated by experimental set are shown. (C) Number of genic barcodes and (D) unique genes represented in all combinations of 1–15 samples when pooled together in silico, separated by sample type. Curves approach saturation at low replication. See S1 Data for numerical values.
(TIF)

S1 Data Supporting information. Included in the S1 Data file are: **"barseq summary data"**: Gene annotation information, RB-TnSeq summary statistics, *in vitro* Fitness Scores for 148 fitness assays previously described [23], operon assignment, and COG information for all 4576 genes considered in this study. "RPL" refers to fitness score comparing "Root" to "NRI" samples, "NRF" refers to fitness scores comparing NRF to NRI samples, and "RPL.NRF" refers to fitness scores comparing "Root" to NRF samples (the latter used to determine significant colonization genes). **"sample metadata"**: metadata used for barcode sequencing, including index information and file associations (corresponding to files in Open Science Framework repository) **"barseq metadata"**: metadata file used for BarSeqR pipeline **"insertion mutant map"**: list of all mapped insertion mutant strains, with barcode and position information. **"strains selected for validation"**: list of all 22 strains selected for luciferase and lacZ validation assays (Fig 3, S6 and S7 Figs), with corresponding barcode, fitness score, and annotation information. **"P. putida homologs"**: List of all colonization genes identified in [21] with corresponding best-hit matches to WCS417r genes. **"Essential genes"**: Top Database of Essential Genes (http://www.essentialgene.org/) BLAST hit and alignment information for all WCS417r genes. **"Fig 2B COG Categories"**: Data underlying Fig 2B **"Fig 3B, S6 Fig Data (Validat'n)"**: Validation data underlying Fig 3B and S6 Fig., including colonization index (CI), the assay performed, and RB-TnSeq summary statsitics." **"Fig 4 Data (Selected heatmap)"**: Heatmap data underlying Fig 4, including fitness scores, the relevent condition presented, and the panel the data is located in. Note: gene identifiers are gene symbols where applicable, and locus identifiers otherwise. **"S5 Fig Data"**: Data underlying S5 Fig (x axis: Mesh ratio; y-axis: Root Intensity). **"S7 Fig Data (Timecourse Valid)"**: Raw data underlying S7 Fig. For panel A, the "Root Fraction Mutant or wild type" is presented (only for data with sysName locus identifiers). For panels B and C, summary data for all samples is presented (Mesh Cells norm. and Root Cells norm.). **"S11 Fig Data"**: Data underlying S11 Fig. COG, cluster of orthologous group; NRF, no root final; NRI, no root initial; RB-TnSeq, randomly barcoded transposon mutagenesis sequencing.
(XLSX)

## Abbreviations

COG: cluster of orthologous group
ISR: induced systemic resistance
Lux+: luciferase-producing
NFI: no root initial
NRF: no root final
PGPR: plant growth-promoting rhizobacteria
RB-TnSeq: randomly barcoded transposon mutagenesis sequencing
TnSeq: transposon mutagenesis sequencing
WT: wild-type
