# Supplementary figures and images for: Genome-wide identification of bacterial plant colonization genes
Source: PLoS Biol. 2017 Sep 22;15(9):e2002860. doi: 10.1371/journal.pbio.2002860 (PMC5627942; doi:10.1371/journal.pbio.2002860)

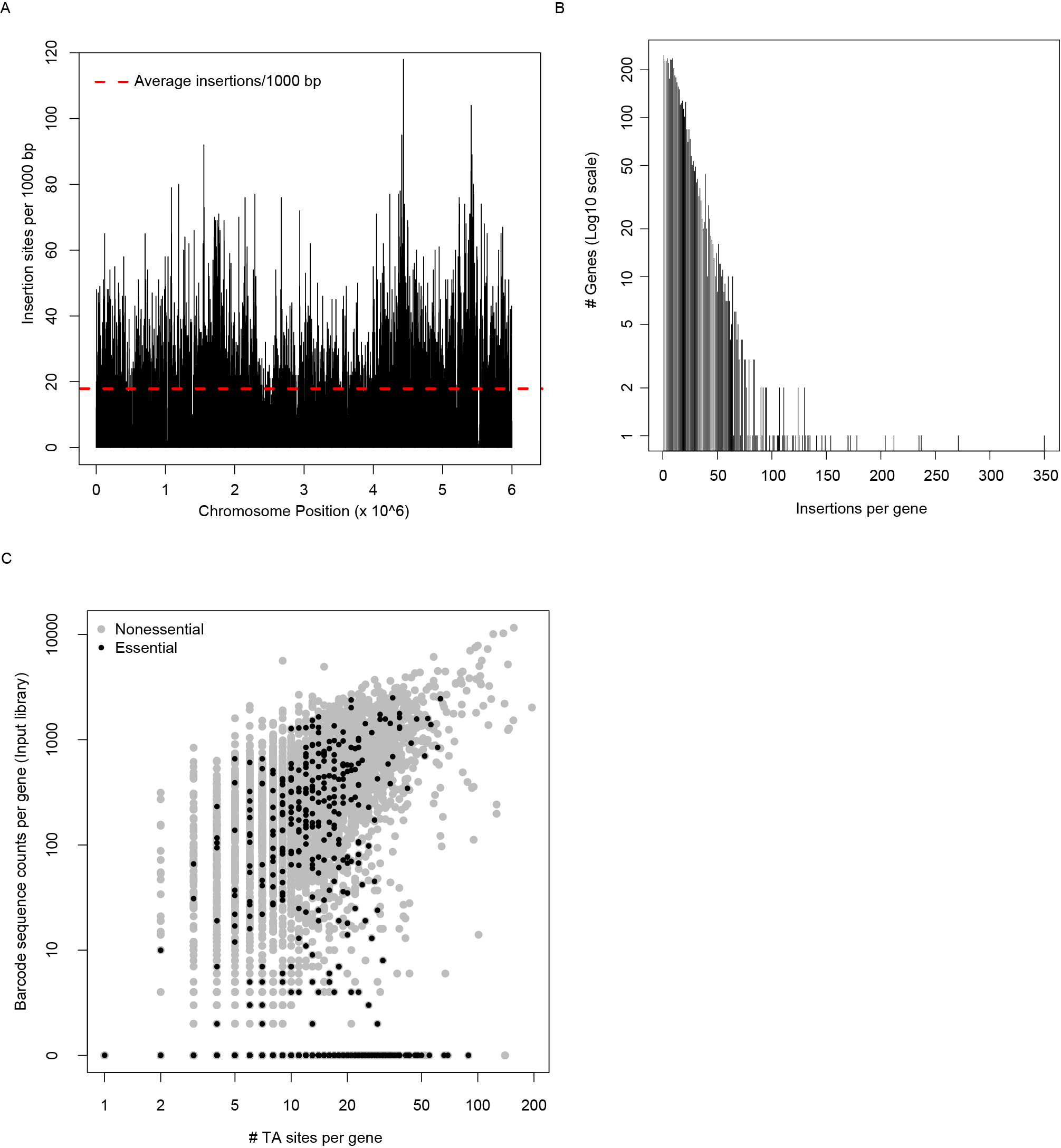

Supplement: S1 Fig — Mariner mutagenesis of P. simiae WCS417r and subsequent TnSeq resulted in a library of 110,142 individual mutant strains with mapped insertion sites and unique barcodes, distributed across the genome at an average rate of 18 insertions per 1000 bp (A). Of the 5,610 genes in the genome, we generated mutations in 4,709, with most genes having fewer than 50 insertions (B). The number of insertions found within a gene is largely a function of the number of thymine-adenine (TA) dinucleotide sites (C). 385 out of the 827 genes with no insertion mutants, and 307 of the 4,783 genes with viable insertion mutant strains represented in the library share significant homology with genes in the Database of Essential Genes (C, black points). TnSeq, transposon mutagenesis sequencing. (TIF) [file pbio.2002860.s001.tif]

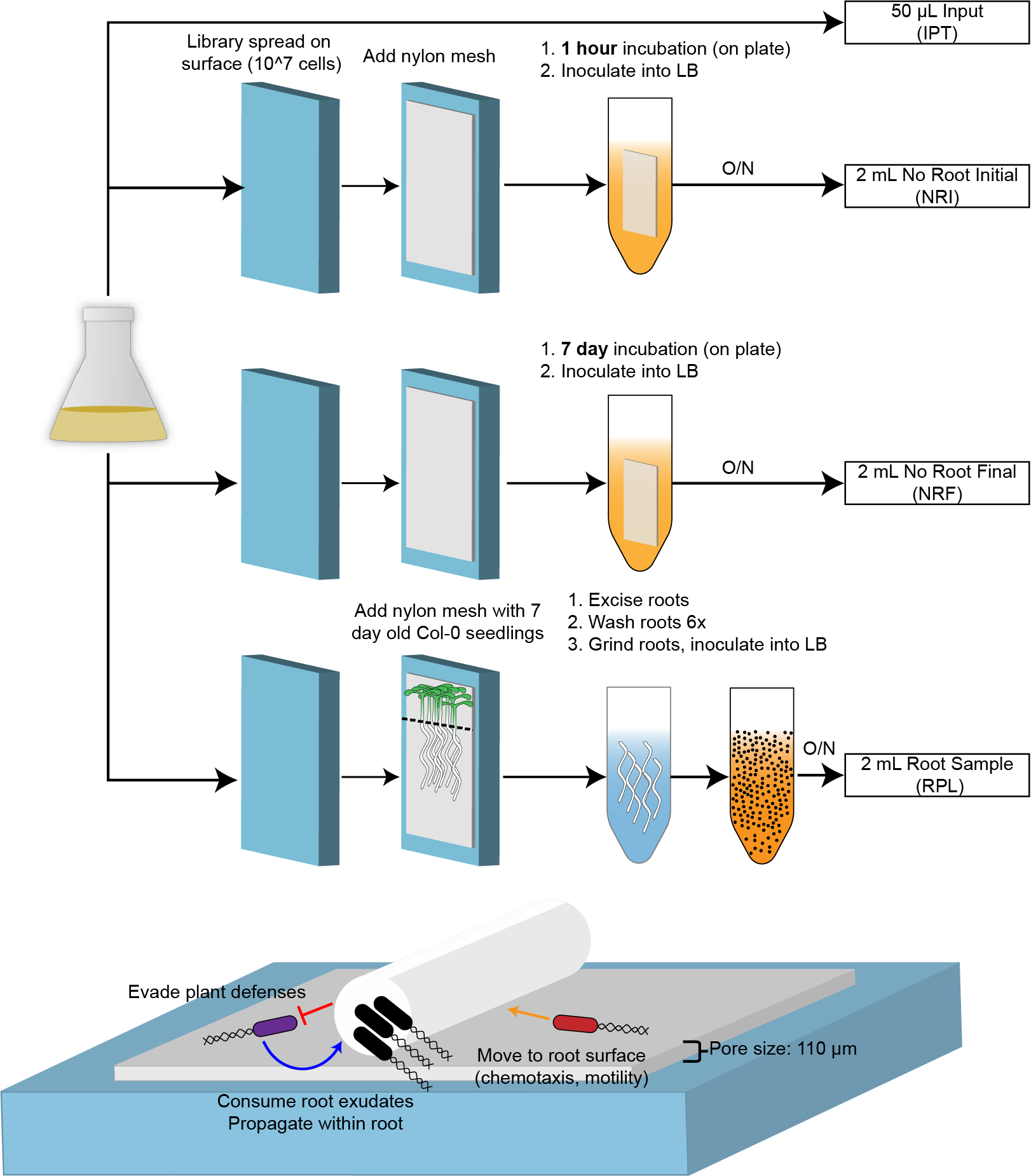

Supplement: S2 Fig — Bacterial samples used for sequence-based identification of colonization genes were collected by inoculating the RB-TnSeq insertion mutant library on nylon mesh supporting Col-0 seedlings, then culturing the colonized roots (excised and washed) in rich media overnight. Control samples also collected for this analysis included an input library control (collected from the inoculation culture), as well as overnight cultures of colonized nylon mesh without plants incubated for 1 hour or 7 days. RB-TnSeq, randomly barcoded transposon mutagenesis sequencing. (TIF) [file pbio.2002860.s002.tif]

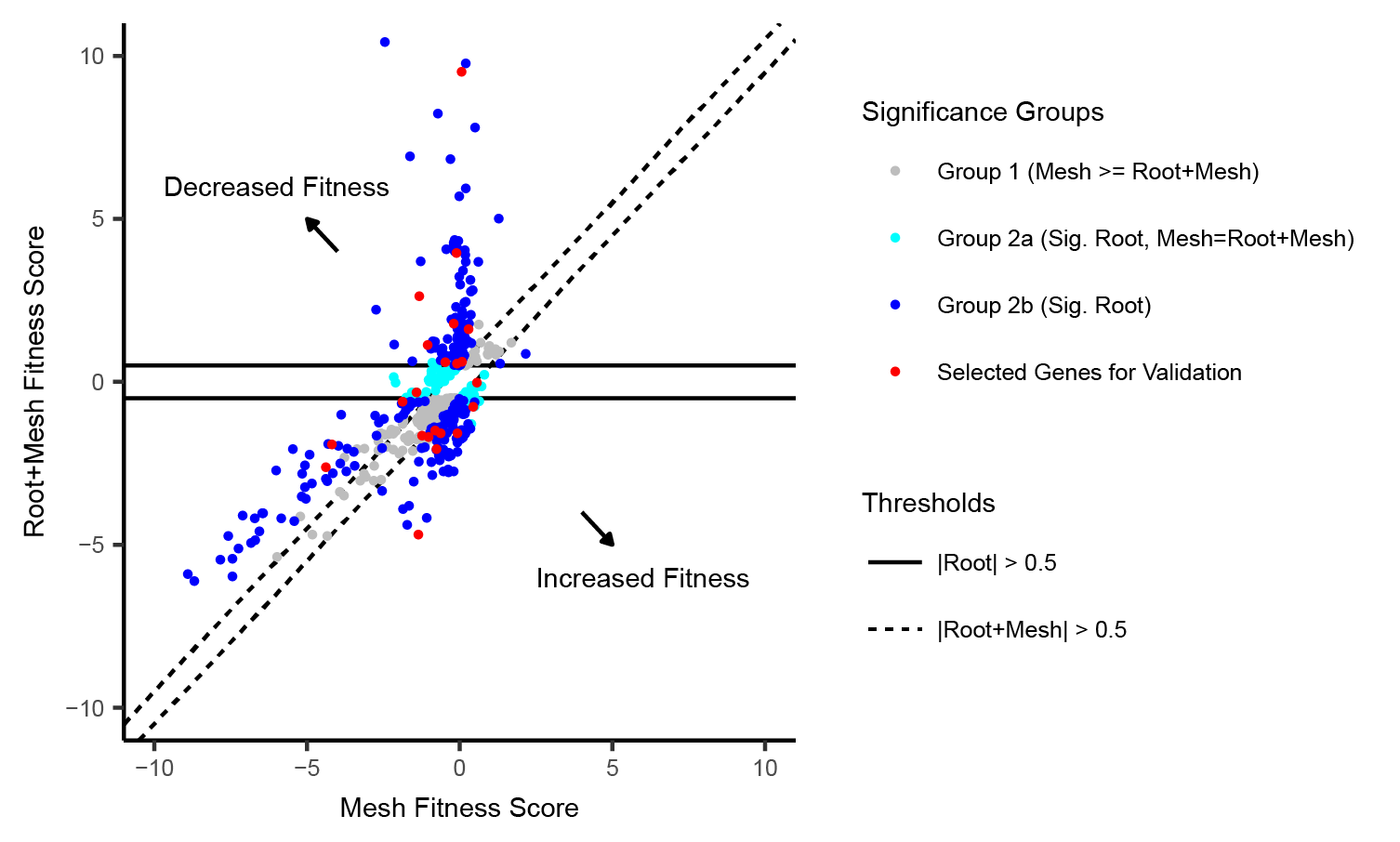

Supplement: S3 Fig — Fitness scores of root-derived mutant strains (normalized to the fitness scores derived from pre-colonization NRI samples) are plotted (y-axis) against fitness scores from post-colonization NRF-derived mutant strains (also normalized to the NRI mesh input samples; x-axis). Points colored in gray correspond to genes that are significantly enriched or depleted between “Root” (RPL) and NRI samples, but not significantly different between RPL and NRF samples (group 1). Points colored in cyan represent genes significantly different between RPL and NRF samples, but not significantly different between RPL and NRI samples (group 2a). Points colored in dark blue correspond to genes significantly different between RPL and NRF samples, as well as between RPL and NRI samples (P < 0.01, effect size > 0.5) (group 2b). The blue- and cyan-colored genes are considered to be significant colonization genes. Genes circled in red are the 22 genes selected for further validation. NRF, no root final; NRI, no root initial. (TIF) [file pbio.2002860.s003.tif]

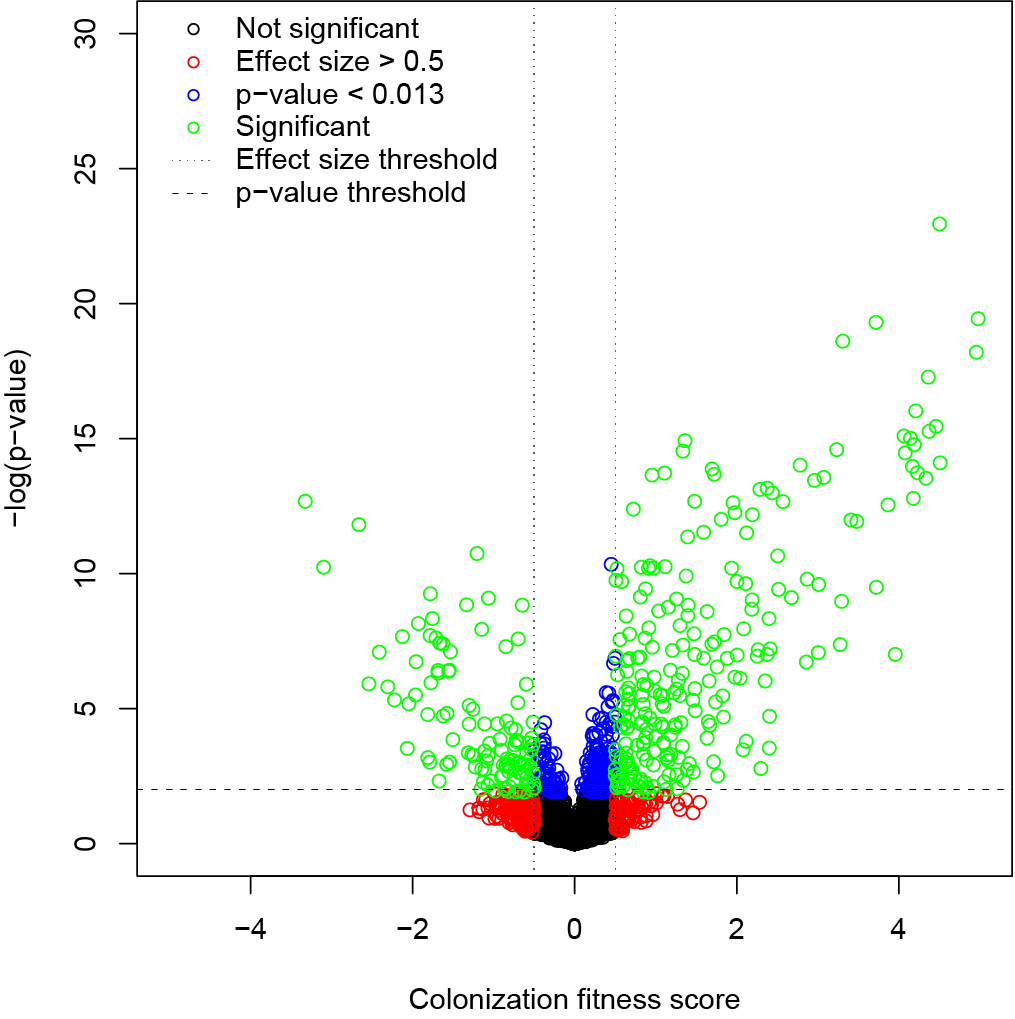

Supplement: S4 Fig — To determine which genes significantly contribute to root colonization, we performed a two-stage thresholding strategy. Genes having P values < 0.013 (t-test; FDR = 0.05; dashed line; genes above shown in blue and green) and having an effect size (absolute value of the fitness score) > 0.5 (dotted line, genes above or below shown in red and green) were determined to be significant (shown in green). Across the 4,576 genes considered in this screen, most fitness values were normally distributed about zero, although the fitness score distribution contained a relatively long high-fitness value tail (upper right quadrant). FDR, false discovery rate. (TIF) [file pbio.2002860.s004.tif]

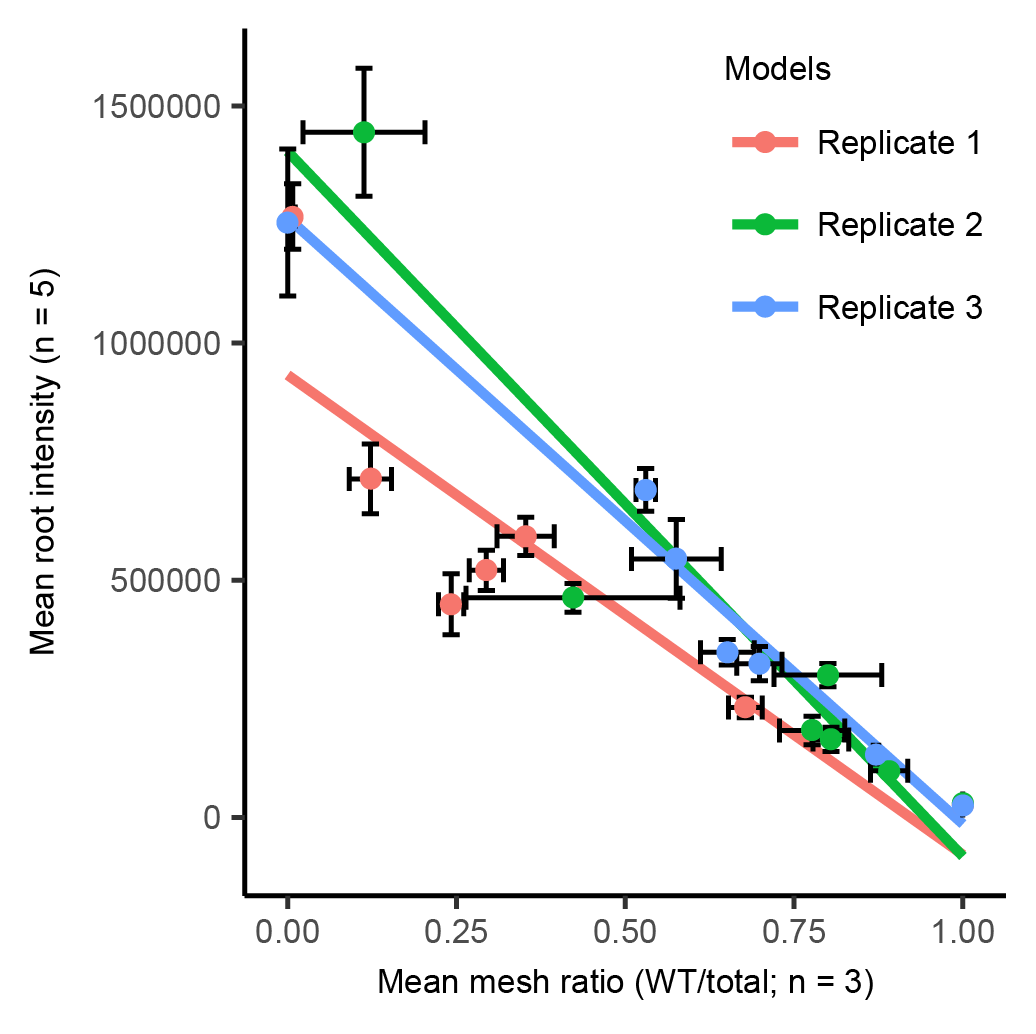

Supplement: S5 Fig — Root intensities of independent competition experiments are plotted against observed WT and Lux+ ratios obtained from inoculated phytagel plates/mesh without roots. Standard curves for three independent assays (corresponding to the three independent biological replicates performed for each insertion mutant strain) are shown. See S1 Data for numerical values. Lux+, luciferase producing; WT, wild-type. (TIF) [file pbio.2002860.s005.tif]

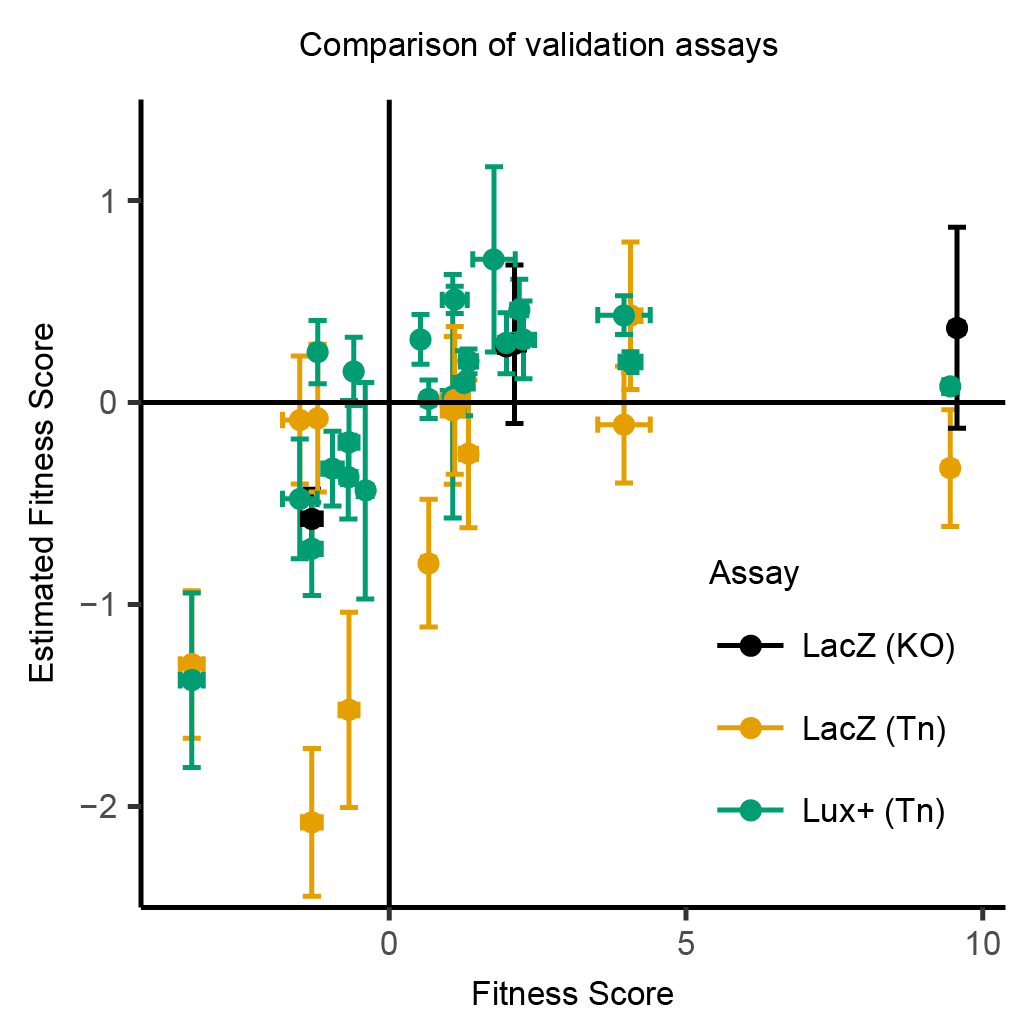

Supplement: S6 Fig — LacZ- and a Lux+ based methods for quantifying competitive colonization ability were performed on a set of insertion mutant strains (S1 Data, Materials and methods). The Lux+ method was performed on all 22 insertion mutant strains (green), the LacZ method was performed on 12 of these 22 mutant strains (orange). In addition, 4 genes were mutated via targeted deletion, and were also assayed using the LacZ method (black). Plotted are the colonization indexes for each assay (y-axis) against the Root fitness score determined by the RB-TnSeq method (Materials and methods). Error bars represent the standard error of the mean (n > = 3 replicates for y-axis, n = 15 for x-axis). Lux+, luciferase producing; RB-TnSeq, randomly barcoded transposon mutagenesis sequencing. (TIF) [file pbio.2002860.s006.tif]

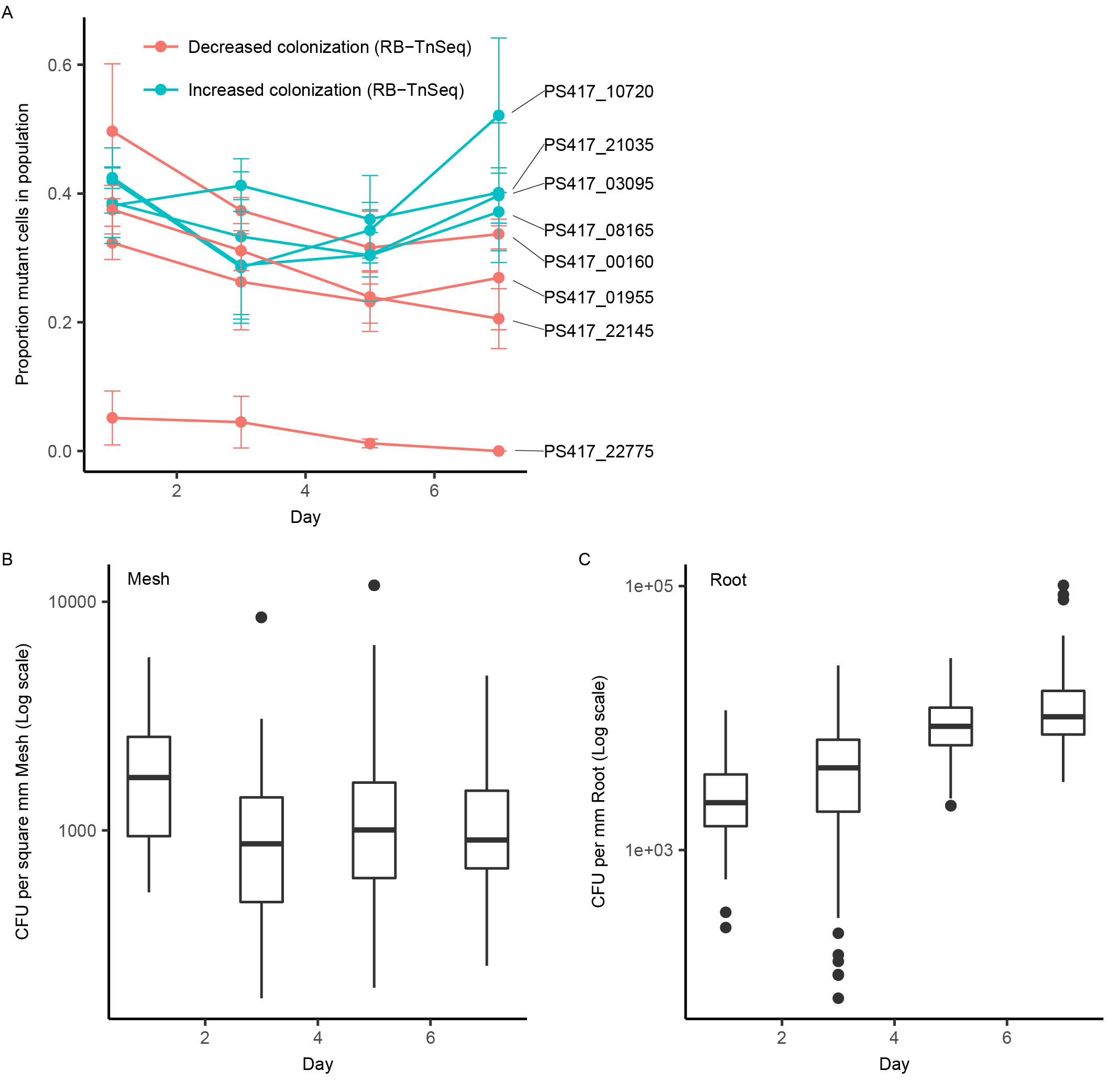

Supplement: S7 Fig — We selected eight mutants and subjected them to a competitive colonization assay with the LuxABCDE expressing P. simiae WCS417r strain as described previously (Fig 3), though harvesting cells for quantification using a sonicating water bath followed by CFU counting for to estimate the ratio of Lux+—and to Lux- + cells from roots following 1, 3, 5, or 7 days after initial inoculation (see Materials and methods). Log 10 total cells recovered from 3 sonicated roots are shown in blue, and the estimated number of cells that are Lux- (i.e. colonization mutant) are shown in red. (A) The fraction of mutant cells in each root population are shown in red (for predicted colonization depleted mutants) and blue (for predicted colonization enriched mutants). Error bars represent the standard error of the mean (n = 3 independent replicates). We also measured total cell counts for each population grown on nylon mesh alone (B) or roots (C) over the same time course, noting the slow decline of bacteria grown on mesh versus the exponential growth of cells grown on roots. See S1 Data for numerical values. CFU, colony forming units; Lux, luciferase. (TIF) [file pbio.2002860.s007.tif]

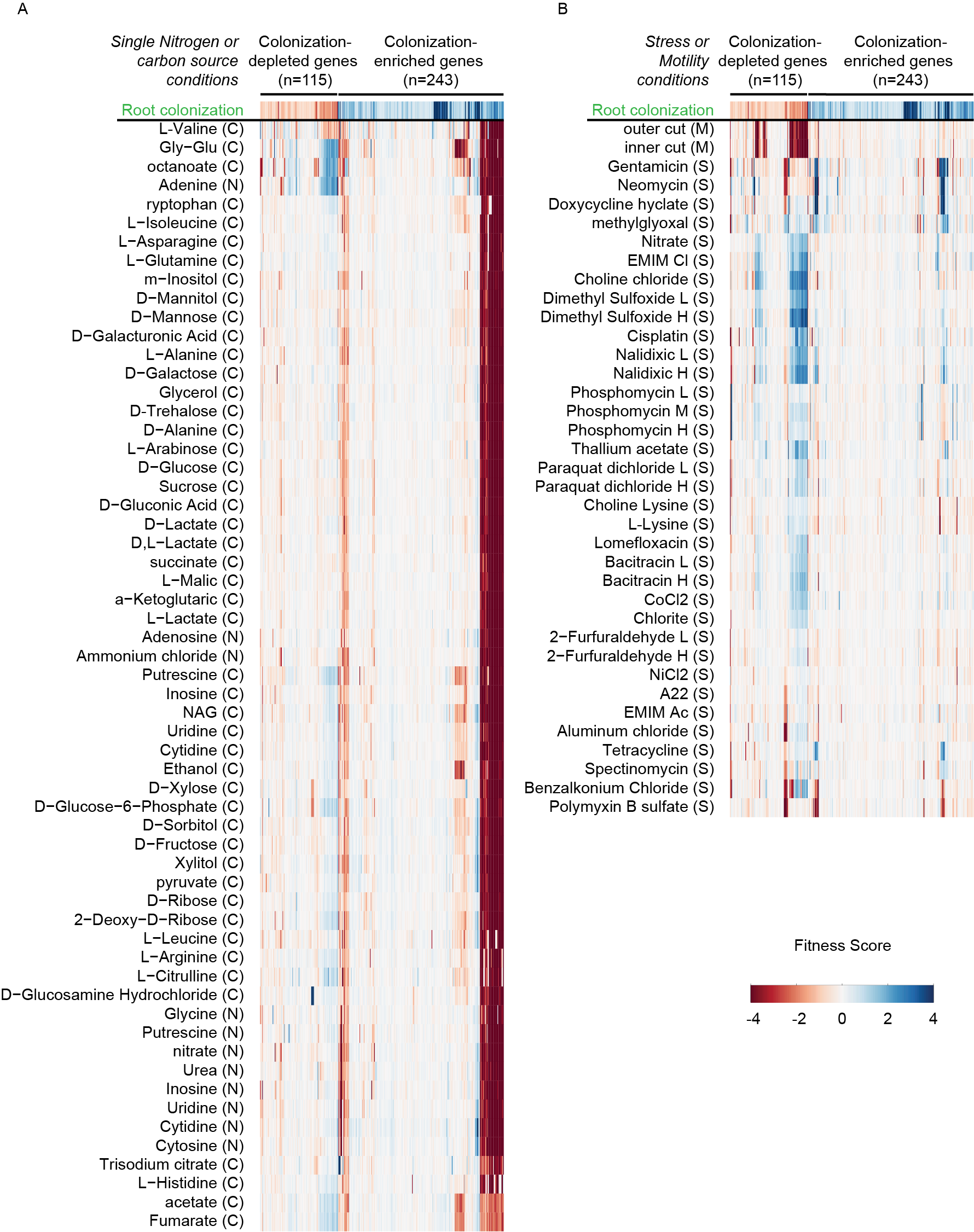

Supplement: S8 Fig — Heatmaps of in vitro fitness data for all colonization genes. All data was clustered row- and column-wise using an unsupervised hierarchical clustering algorithm (R hclust package), re-ordered to separate colonization-depleted and enriched genes (preserving the clustering order within each category), and then split to separate single carbon/nitrogen sources (A) and motility or stress conditions (B). Color scale is shown at the bottom right. Data used to generate these heat maps is included in S1 Data. (TIF) [file pbio.2002860.s008.tif]

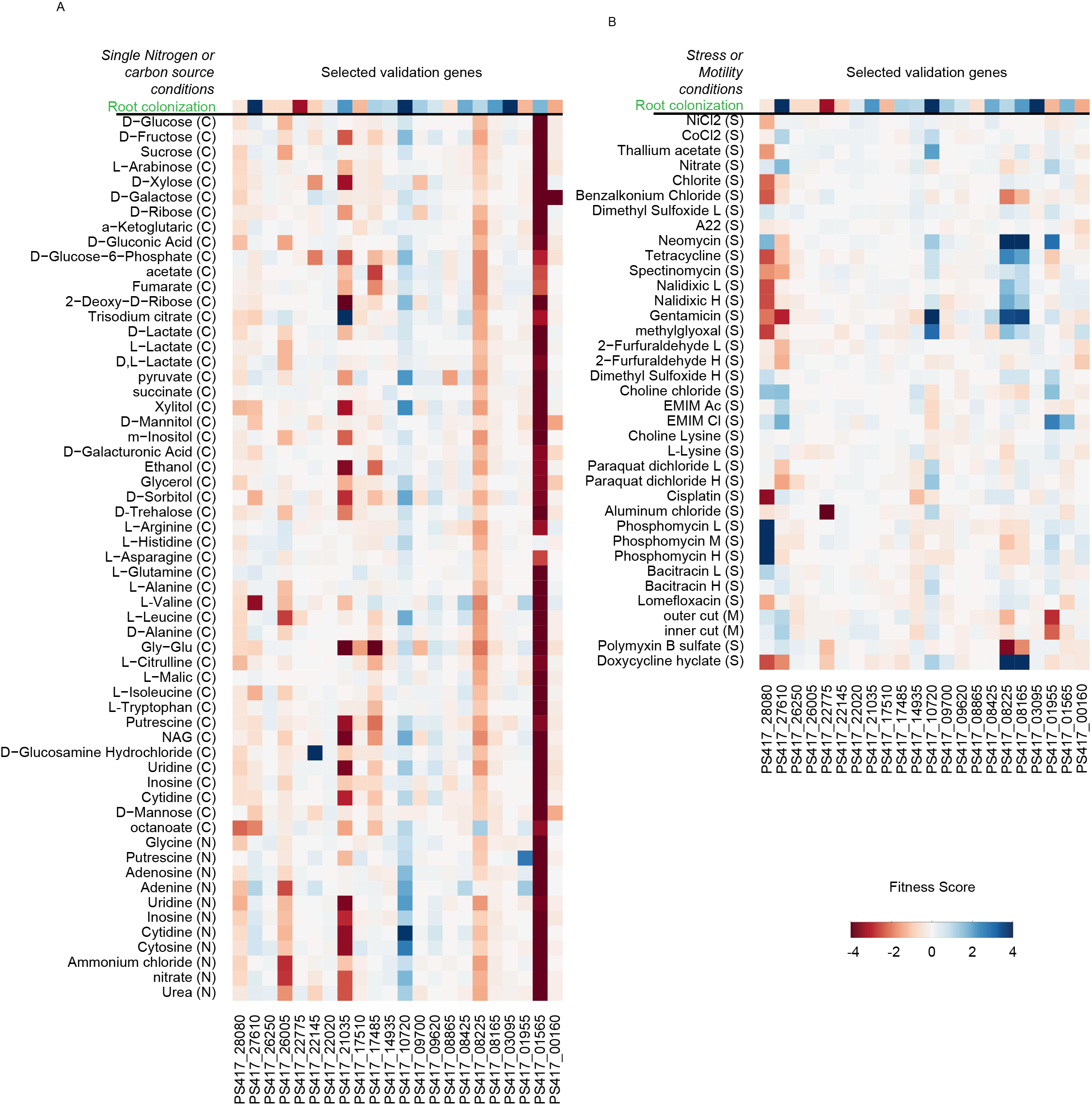

Supplement: S9 Fig — 22 insertion mutants were selected based on their predicted function or the severity of their defect in colonizing the root. Heatmaps (unclustered) of in vitro and root fitness data for these 22 colonization genes are split to separate single carbon/nitrogen sources (A) and motility or stress conditions (B). Color scale is shown at the bottom right. Data used to generate these heat maps is included in S1 Data. (TIF) [file pbio.2002860.s009.tif]

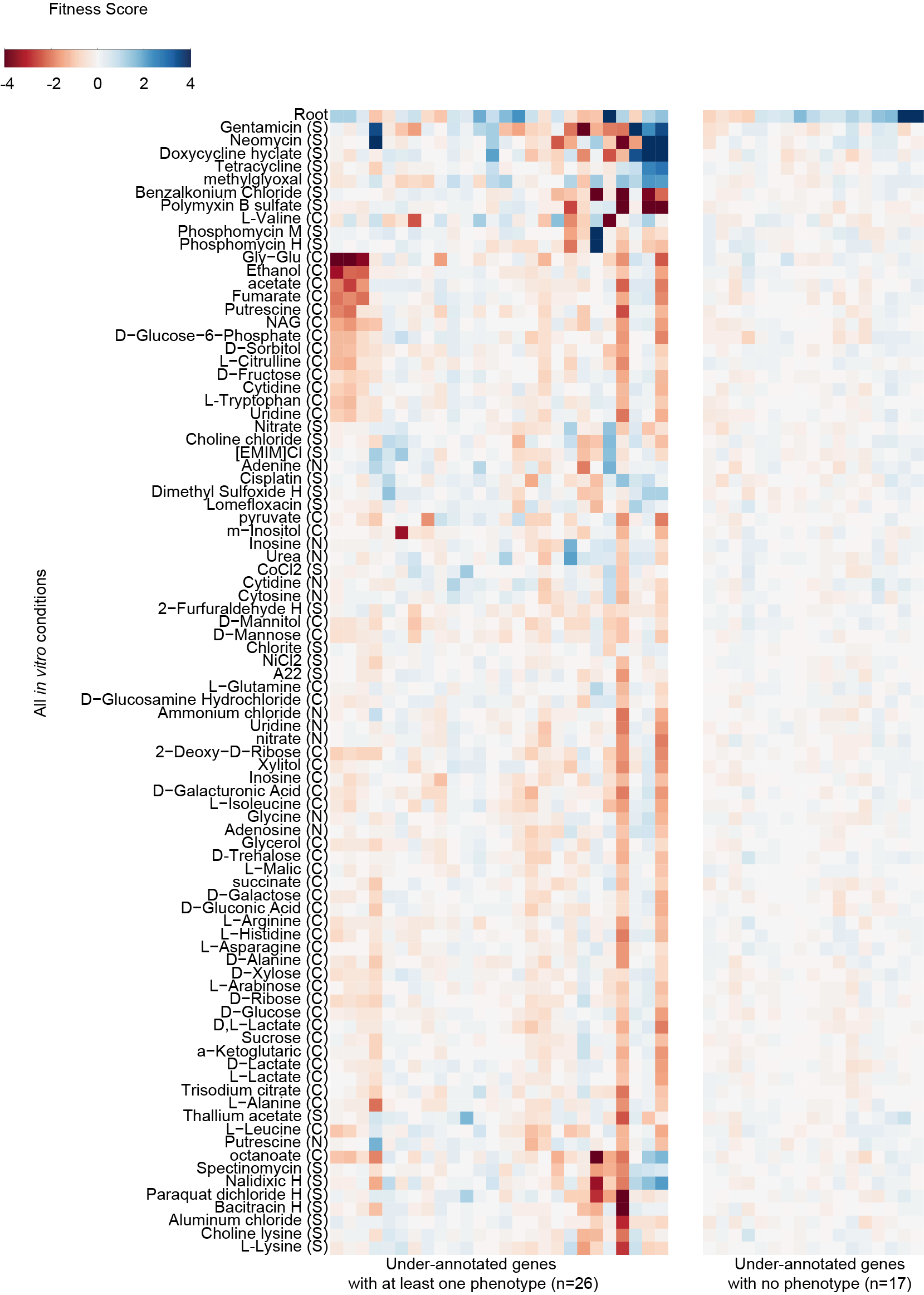

Supplement: S10 Fig — 44 Under-annotated genes with significant root colonization fitness scores were selected based on their COG category (No COG annotation, General Function Prediction Only, Unknown Function) or gene description (hypothetical protein). In vitro and root fitness scores for all conditions are shown here. The data was clustered row- and column-wise using an unsupervised hierarchical clustering algorithm (R hclust package) and then split to separate genes with a phenotype (|fitness score| > 1) in any one in vitro condition (left) or genes with no apparent phenotype under any in vitro condition (right). Color scale is shown at the top left. Data used to generate these heat maps is included in S1 Data. COG, cluster of orthologous group. (TIF) [file pbio.2002860.s010.tif]

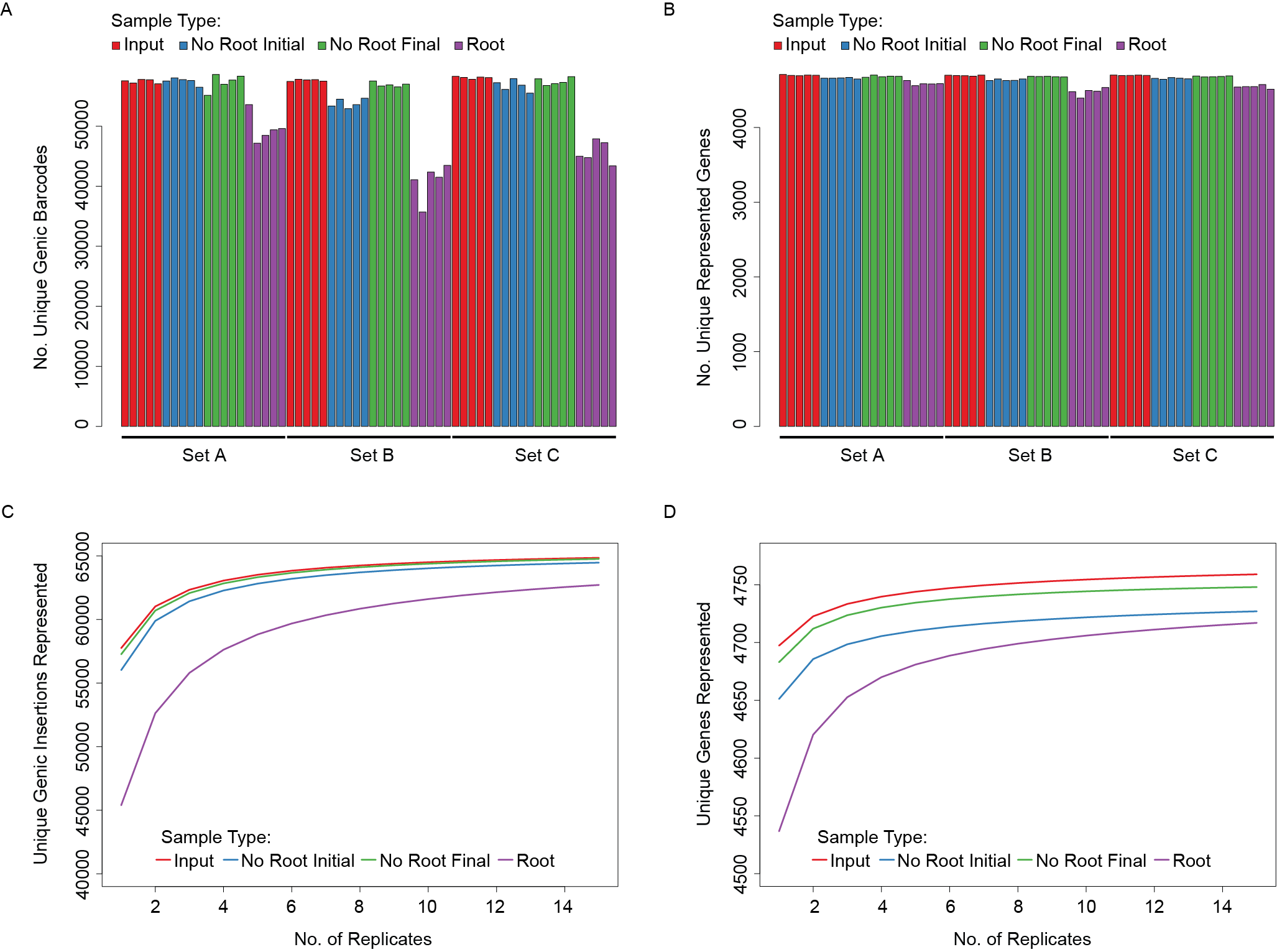

Supplement: S11 Fig — Barcode and gene insertion recovery metrics. Number of (A) unique genic barcodes (associated with insertion sites between 10% and 90% of a gene body) recovered and (B) gene knockouts represented from 5 replicates of each sample type, separated by experimental set are shown. (C) Number of genic barcodes and (D) unique genes represented in all combinations of 1–15 samples when pooled together in silico, separated by sample type. Curves approach saturation at low replication. See S1 Data for numerical values. (TIF) [file pbio.2002860.s011.tif]
